# Regional to tertiary inter-hospital transfer versus in-house percutaneous coronary intervention in acute coronary syndrome

**DOI:** 10.1371/journal.pone.0198272

**Published:** 2018-06-21

**Authors:** Delara Javat, Clare Heal, Jennifer Banks, Stefan Buchholz, Zhihua Zhang

**Affiliations:** 1 Department of Cardiology, Mackay Base Hospital, Mackay, QLD, Australia; 2 Mackay Clinical School, School of Medicine and Dentistry, James Cook University, Mackay Campus, Mackay, QLD, Australia; 3 Mackay Institute for Research and Innovation (MIRI), Mackay, QLD, Australia; 4 HeartCare Western Australia, Suite 21, St John of God Hospital, Southwest Health Campus, Bunbury, WA, Australia; IRCCS Policlinico S.Donato, ITALY

## Abstract

**Rationale:**

To address the inaccessibility of interventional cardiac services in North Queensland a new cardiac catheterisation laboratory (CCL) was established in Mackay Base Hospital (MBH) in February 2014.

**Objective:**

To determine whether the provision of in-house angiography and/or percutaneous coronary intervention (PCI) 1) minimises treatment delays 2) further reduces the risk of mortality, recurrent myocardial infarction (MI) and recurrent ischaemia 3) improves patient satisfaction and 4) minimises cost expenditure compared with inter-hospital transfer for patients with acute coronary syndrome (ACS).

**Methods:**

We compared ACS patients who were transferred to tertiary centres from July 2012 to June 2013 with those who received in-house angiography and/or PCI from February 2015 to January 2016. The primary outcome was the composite of all-cause mortality, recurrent myocardial infarction (MI) or recurrent ischaemia at six months. Pre-specified secondary outcomes were the composite of all-cause mortality, recurrent MI or recurrent ischaemia at one month, a summated patient satisfaction score and the proportional cost savings generated between 2015 and 2016.

**Results:**

We included consecutive samples of 203 patients from July 2012 to June 2013 and 229 patients from February 2015 to January 2016. There was a reduction in the median time to treatment of 3.2 days and a reduction in the median length of stay of four days amongst all ACS patients receiving in-house angiography and/or PCI. The primary outcome occurred in 14 (6.9%) patients in the 2012 to 2013 group, as compared with 18 (7.9%) patients in the 2015 to 2016 group (OR = 0.71, 95% CI 0.24–2.1, P = 0.54). The secondary outcome at one month occurred in four (2.0%) patients in the 2012 to 2013 group, as compared with three (1.3%) patients in the 2015 to 2016 group (OR = 1.2, 95% CI 0.11–13.1, P = 0.87). There was a statistically significant improvement in the summated patient satisfaction score amongst patients who received in-house angiography and/or PCI (U = 1918, P <0.05 two tailed). A calculation of estimated cost savings showed a reduction in proportional cost of $14 481 (51%) per ACS patient receiving in house angiography and/or PCI between 2015 and 2016.

**Conclusion:**

This study suggests that the provision of regional in-house angiography and/or PCI for the treatment of ACS minimises delays to invasive treatment by 3.2 days, minimises the median length of stay by four days, significantly improves patient satisfaction and reduces proportional treatment costs by $14 481 (51%) per patient. Currently, however, it appears that that in-house treatment does not further reduce the risk of mortality, recurrent MI and recurrent ischaemia at one and six months.

## Introduction

Cardiovascular diseases are currently the leading cause of deaths globally.[1] Amongst these, coronary artery disease (CAD) is the single leading cause for mortality in Australia.[2] Acute coronary syndrome (ACS) is a manifestation of CAD which includes ST elevation myocardial infarction (STEMI) and non-ST elevation acute coronary syndrome (NSTEACS). Current Australian ACS guidelines recommend invasive management with diagnostic angiography and/or percutaneous coronary intervention (PCI) for all STEMI and high risk NSTEACS patients.[3] This is based on recent literature which demonstrates superior long term outcomes with primary PCI compared to in-hospital fibrinolysis for STEMI patients.[4] Furthermore randomised controlled trials demonstrate a long-term mortality benefit at one and five years with a routine invasive strategy compared with a conservative approach for high risk NSTEACS patients.[5, 6] Further research also showed a reduction in major cardiac events amongst patients randomised to an early invasive strategy with concomitant glycoprotein IIb/IIIa treatment.[7] Unfortunately, compared to their metropolitan counterparts, regional communities in Australia are frequently characterised by geographical isolation, limited access to advanced medical facilities and protracted treatment delays.[8] As such regional populations may be at higher risk of adverse outcomes following an acute coronary event.[9] In particular, the provision of cardiac catheterisation remains a challenge in North Queensland, where more than 50% of the population resides outside the state’s capital.[10]

To address the inaccessibility of interventional cardiac services in North Queensland a new cardiac catheterisation laboratory (CCL) was established in Mackay Base Hospital (MBH) in February 2014. Angiography became immediately available, whilst PCI became routinely available from February 2015. Prior to the establishment of the CCL all ACS patients presenting to MBH relied on inter-hospital transfer to tertiary cardiac centres in Townsville and Brisbane for expedited management. Since the establishment of the CCL research has not yet been conducted locally to assess the clinical, social and financial impact of service on the management of ACS. Furthermore studies comparing inter-hospital transfer and in-house catheterisation are lacking. In view of this we conducted a single-centre study comparing the model of regional to tertiary inter-hospital transfer with in- house angiography and/or PCI with respect to treatment delays, clinical outcomes, patient satisfaction and cost for ACS patients presenting to MBH.

## Methods

### Aim and research questions

The aim of our research was to compare patients who were transferred to tertiary centres for angiography and/or PCI between 2012 and 2013 with patients who received invasive treatment in MBH from 2015 to 2016. We aimed to determine whether the provision of in-house angiography and/or PCI 1) minimised treatment delays 2) further reduced the risk of mortality, recurrent myocardial infarction (MI) or recurrent ischaemia 3) improved patients satisfaction and 4) minimised cost expenditure for patients with ACS presenting to MBH.

To address the aim we designed three research questions, 1) “Was the risk of death, reinfarction and recurrent ischaemia further reduced at one month and six months amongst ACS patients who received angiography and/or PCI regionally between 2015 and 2016 compared with those transferred for treatment between 2012 and 2013?”, 2) “Was patient satisfaction increased when ACS patients received regional angiography and/or PCI?” and 3) “What was the estimated cost saving associated with in-house angiography and/or PCI for the treatment of regional ACS patients between 2015 and 2016?” The methods for the project were specified in advance and documented in a research protocol.

### Study design

Our study design consisted of three parts. Retrospective clinical audit assessed the clinical outcomes at one and six months with additional telephone interviews with patients with missing clinical information. Secondly, a patient satisfaction survey was sent to eligible participants. To assess cost expenditure we identified sources of cost savings for ACS patients treated regionally between 2015 and 2016 and compared these with the actual in-patient costs for the same year.

Across the study, patients were categorised into two groups according to the location of their angiogram and/or PCI. The first group included a consecutive sample of all ACS patients who were admitted to MBH Coronary Care Unit (CCU) from 1^st^ July 2012 to 30^th^ June 2013 and were subsequently transferred to a tertiary centre for an angiogram and/or PCI. The second group of patients included a consecutive sample of ACS patients who were admitted to MBH CCU from 1^st^ February 2015 to 31^st^ January 2016 and received an inpatient angiogram and/or PCI in the same hospital.

The Townsville Hospital and Health Service Human Research Ethics Committee (HREC) approved the study and waived the need for informed consent (Reference number HREC/15/QTHS/89).

### Eligibility criteria

Patients were eligible for inclusion if they were: 1) diagnosed with ACS as per Australian Guidelines and 2) received an inpatient angiogram and/or PCI, within the same admission.[3] Acute coronary syndrome was defined as either unstable angina (angina worsening in severity or frequency, or lowering of angina threshold), NSTEMI (clinical features consistent with ACS and any of: persistent or dynamic ST-segment depression ≥ 0.5mm or new T wave inversion ≥ 2 mm) or STEMI (clinical symptoms consistent with ACS and any of: persistent ST segment elevation of ≥1mm in two contiguous leads, ST-segment elevation ≥2mm in two contiguous chest leads or new left bundle branch block (LBBB).

Patients were ineligible if they 1) were diagnosed with stable angina, 2) were referred for an angiogram and/or PCI secondary to a positive stress test, 3) received medical management only or 4) were treated with an elective outpatient angiogram and/or PCI.

### Outcomes

#### Primary outcome

The primary clinical outcome was the first occurrence of the composite of all-cause mortality, recurrent MI or recurrent ischaemia at six months after the inpatient angiogram and/or PCI. Recurrent MI was defined as a clinical diagnosis of NSTEMI or STEMI as per Australian guidelines occurring more than 48 hours after the incident MI.[3] Recurrent ischaemia was defined as recurrent ischaemic symptoms whilst the patient was on optimal medical therapy with or without documented electrocardiogram (ECG) changes indicative of ischaemia and/or an indication for additional intervention.

#### Secondary outcomes

Secondary outcomes were:

1. Composite of all-cause mortality, recurrent MI or recurrent ischaemia at one month
2. Major bleeding at one and six months as defined by the Thrombolysis in Myocardial Infarction (TIMI) criteria for major non- coronary artery bypass graft (CABG) related bleeding.[11]
3. Patient satisfaction as measured by a summated patient satisfaction score.
4. Estimated proportional cost saving for ACS patients treated regionally between 2015 and 2016.

See Supporting Information (S1 Appendix) for the definition of Major TIMI bleeding.

### Data collection

#### Retrospective clinical audit

Data for the retrospective clinical audit were extracted from electronic and hard copy patient medical records.

The following participant characteristics were recorded for all patients: age, gender, smoking status, body mass index (BMI) and co-morbidities (hypertension, hyperlipidaemia, diabetes mellitus, chronic kidney disease, positive family history, previous MI, previous PCI, previous CABG, past stroke). Diagnostic characteristics were also recorded for all patients including, location of ischaemia or infarction on ECG (anterior, lateral, inferior, posterior or non-specific), MI complications (pulmonary oedema, shock and high degree heart block), peak troponin level and ejection fraction. Additionally two mortality scores were recorded, the TIMI score and the Global Registry of Acute Coronary Events (GRACE) score. (The components of both scores are detailed in the Supporting Information S1A–S1C Table).

The following treatment characteristics were recorded for each patient: the name of the tertiary accepting hospital, the procedure at the tertiary accepting hospital, the procedure at MBH, clopidogrel loading, ticagrelor loading and discharge medications. Procedural information regarding angiography was recorded including, the site of access (radial or femoral approach), catheter gauge, stent type, infarct related artery (artery with ≥ 70% stenosis), TIMI flow pre -and post- PCI, number of vessels with ≥70% stenosis, number of vessels treated and the number of stents used.

Time to transfer was defined as the number of days from admission to MBH until admission at the tertiary accepting hospital for patients transferred for treatment. Time to treatment was defined as the number of days from admission to MBH until the angiogram and/or PCI. The total length of stay was defined as the time from day of admission to MBH until day of discharge from the tertiary hospital or MBH as applicable.

Where a patient underwent an angiogram and a subsequent CABG, only the time to treatment and procedural characteristics of the angiogram were recorded.

Data were collected regarding the rate of mortality, recurrent MI, recurrent ischaemia and major TIMI bleeding at one and six months. Information was gathered from the follow-up telephone interviews routinely conducted by the MBH CCL nurses at one month between 2015 and 2016.

#### Telephone interview

An interview pro-forma was designed to collect information from patients regarding clinical outcomes where data were unavailable in hospital records. The pro-forma is available in the Supporting Information (S2 Appendix).

#### Patient satisfaction survey

A satisfaction survey was employed to measure the construct of patient satisfaction. The questionnaire was specifically designed and modelled upon previously validated patient satisfaction surveys.[12] Satisfaction was rated with the length of time waiting for their procedure, mode of transport for inter-hospital transfer and overall convenience of the procedure. A five-point Likert-type response scale of one = very dissatisfied, two = dissatisfied, three = neither dissatisfied nor satisfied, four = satisfied and five = very satisfied was used. Additional comments were invited at the end of the survey regarding patient experience. All material and correspondence was anonymous. The patient information leaflet and survey are available in the Supporting Information (S3A and S3B Appendix).

#### Estimated cost savings

Cost savings were ascribed to the CCL reducing the need for inter-hospital transfer and shortening delays to treatment. Specifically we attributed saving to 1) patients and escorts avoiding travel, 2) escorts avoiding overnight accommodation and 3) a reduction in the total length of stay and corresponding number of bed-days.

The cost of the Royal Flying Doctor Service (RFDS) and the Queensland Ambulance Service (QAS) per patient was collected from the Aero Medical Retrieval and Disaster branch of the hospital. The cost of escort travel, patient return and the bed-day cost for MBH CCU beds between 2015 and 2016 were retrieved from the accounting department.

The estimated savings from avoiding travel to the tertiary hospital were calculated by multiplying the cost of one RFDS and two QAS journeys (one to the Mackay Airport and another to the accepting hospital) by the number of ACS patients that avoided inter-hospital transfer between 2015 and 2016. The cost of escort travel to Townsville and escort and patient return to Mackay by car, as subsided by the Queensland Health Patient Travel Subsidy Scheme (PTSS), was multiplied by the number of ACS patients that avoided inter hospital transfer.

Cost for overnight accommodation was calculated as required for all ACS patients who avoided inter- hospital transfer between 2015 and 2016. Furthermore, treating patients regionally potentially led to earlier discharge. We calculated the cost of one bed-day in MBH CCU and multiplied this by any reduction in median length of stay for all ACS patients who avoided inter-hospital transfer.

Additionally we obtained actual inpatient costs for all ACS patients treated regionally between 2015 and 2016 by summing the costs related to each patient’s diagnosis related group (DRG). Finally we calculated the estimated the cost saving per patient treated from 2015 to 2016 as a percentage of potential cost with inter-hospital transfer included.

Costs excluded were 1) the establishment and maintenance of the regional catheterisation laboratory and 2) indirect benefits such as a reduction in bed pressure at the accepting hospital. Conservative values were used for all parts of the cost calculation.

Data for all three parts of the study were extracted into a pre-designed form which was piloted on ten patients and refined accordingly. Data were extracted by an independent researcher and reviewed 24 hours after completed extraction to check for errors.

### Statistical analysis

IBM SPSS Statistics Version 22.0 software was used. We assumed an event rate for the primary outcome of 17% in the 2012 to 2013 group and 9% in the 2015 to 2016 group based on previous literature.[13] Using a difference of two proportions, the sample size was 274 patients per group with a power of 80% and an alpha level of 0.05.

Descriptive analyses were carried out with categorical data were expressed as counts and frequencies and continuous and ordinal data expressed as median and interquartile ranges. All decimal values were rounded to two significant figures. We did not impute missing outcome data for any outcomes.

Univariate logistic regression analyses were performed to explore the relationships in the data between the independent variables and the composite outcomes of mortality, recurrent MI or recurrent ischaemia at one and six months. To avoid spurious significant p values only the following independent variables were analysed: age, male gender, comorbidities (hypertension, hyperlipidaemia, diabetes, chronic kidney disease, BMI), smoker, positive family history, previous MI, previous PCI, previous CABG, past stroke, time to procedure, year of admission, mortality scores (TIMI and GRACE), three or more vessels with ≥70% stenosis) and antiplatelet use (clopidogrel loading and ticagrelor loading). A p-value <0.05 was considered significant.

Multivariate logistic regression analyses were performed to control for confounding factors. We focused on the causal effect of the year of admission therefore this independent variable was included in the final multivariate regression model as the ‘exposure’ variable. Other independent variables were simultaneously included in the binomial logistic regression model because they might have been confounders, rather than being of direct interest. The literature was consulted to further select a smaller set of clinically appropriate confounding variables that would be supported by our sample size. Independent variables included in the final multivariate logistic regression model were: age, sex, hypertension, diabetes, smoking status, previous MI, previous PCI, previous CABG, time to procedure, year of admission, GRACE score, past stroke, three or more vessels with ≥70% stenosis, clopidogrel loading and ticagrelor loading. All selected confounding variables were included in the regression, regardless of their statistical significance on univariate analysis, to ensure they were controlled for in the final model. Automated selection procedures were not relied upon to make decisions about confounders as such methods may result in inappropriate exclusion or inclusions into the final model based on statistical significance. This approach has been previously documented.[14] The odds ratio (OR) was selected as the outcome measure. A p-value of <0.05 was considered significant.

We assessed goodness-of-fit of the fitted model using, the Omnibus test of model, adjusted generalised R^2^ and the Hosmer- Lemeshow goodness-of-fit test. Cases with ZResid values >2.5 or <-2.5 were considered outliers and reconsidered before the final regression analysis. The process was completed for the composite of all-cause mortality, recurrent MI or recurrent ischaemia at one month and at six months.

Separate analyses were conducted for data from the patient satisfaction surveys. Summated score analysis and single item analysis were both performed to analyse the quantitative data because single item analysis alone is an unreliable to measure a single construct.[15] Internal consistency of the scale was measured using the Cronbach’s alpha coefficient, aiming for > 0.8. Items were removed from the summated score analysis where their exclusion increased the internal consistency of the scale. Categorical variables from the patient satisfaction survey were compared by Fisher’s exact test. After assessing for normality with the Shapiro Wilk test ordinal and continuous variables from the survey were compared with non-parametric tests (Mann-Whitney U). A two-tailed p-value <0.05 was considered significant.

A thematic approach was adopted to analyse the comments provided in the patient satisfaction surveys. To maintain transparency and ensure author interpretation was reliable, direct quotations were selected to verify the themes extracted.

## Results

### Retrospective clinical audit

**Fig 1:**
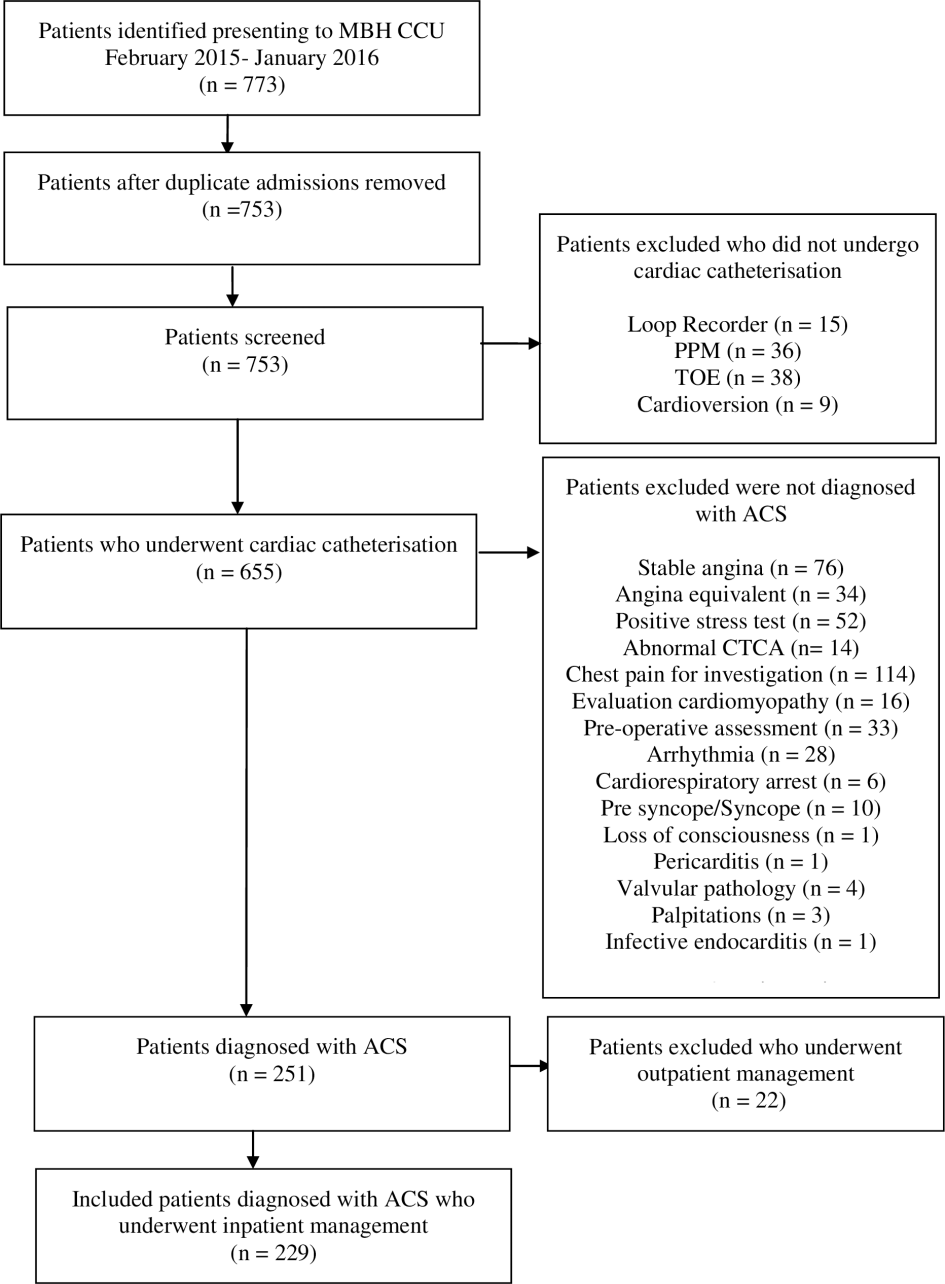
Summary of selection process for patients from February 2015 to January 2016.

A consecutive sample of 203 patients was included from July 2012 to June 2013. We identified 773 patients who presented to the MBH CCL from February 2015 to January 2016 (Fig 1). After duplicate presentations were excluded, we screened 753 patients. We excluded 98 patients because they did not undergo angiography and/or PCI. Of the remaining 655 patients who did undergo cardiac catheterisation we included 229 patients who were diagnosed with ACS and received an inpatient angiogram and/or PCI. A total of 432 patients were included in the retrospective clinical audit. Telephone interviews were conducted with 50 patients from both groups to elicit follow-up information.

#### Participant characteristics

Information regarding the participant characteristics is provided in Table 1. The median age of patients was 62 years. There were 127 (63%) patients who smoked in the 2012 to 2013 group, compared with 67 (29%) in the 2015 to 2016 group. There were 47 (23%) patients with diabetes mellitus in the 2012 to 2013 group, compared with 74 (32%) in the 2015 to 2016 group. Positive family history was present in 49 (24%) patients in the 2012 to 2013 group, compared with 109 (48%) in the 2015 to 2016 group. Previous MI and PCI was recorded in 49 (24%) and 31 (15%) of patients respectively in the 2012 to 2013 group, compared with 52 (23%) and 34 (15%) in the 2015 to 2016 group.

**Table 1:** Patient characteristics for 2012 to 2013 and 2015 to 2016.

Variables	All patients (n = 432)	2012–2013 (n = 203)	2015–2016 (n = 229)
Age y (IR)	62 (18)	60 (17)	65 (19)
Male, n (%)	288 (67)	136 (67)	152 (66)
Smoker, n (%)	194 (45)	127 (63)	67 (29)
Body Mass Index kg/m^2^, median (IR)	28 (8.6)	28 (9.2)	28 (8.4)
Comorbidities, n (%)			
Hypertension	269 (62)	119 (59)	150 (66)
Hyperlipidaemia	259 (60)	120 (59)	139 (61)
Diabetes mellitus	121 (28)	47 (23)	74 (32)
Chronic kidney disease	29 (6.7)	12 (5.9)	17 (7.4)
Positive family history	158 (37)	49 (24)	109 (48)
Previous MI	83 (19)	31 (15)	52 (23)
Previous PCI	65 (15)	31 (15)	34 (15)
Previous CABG	38 (8.8)	17 (8.4)	21 (9.2)
Previous Stroke	11 (2.5)	2 (1.0)	9 (3.9)

**IR-** Interquartile range

**MI**–Myocardial infarction

**PCI**–Percutaneous coronary intervention

**CABG–**Coronary artery bypass graft surgery

#### Diagnostic characteristics

Information regarding the diagnostic characteristics is provided in Table 2. Unstable angina was recorded in 31 (15%) patients in the 2012 to 2013 group and in 86 (38%) patients in the 2015 to 2016 group. STEMI was recorded in 54 (27%) patients from 2012 to 2013, compared with 30 (13%) patients from 2015 to 2016. The median TIMI and GRACE scores were similar between cohorts. Pulmonary oedema was recorded in 16 (7.9%) patients in the 2012 to 2013 group, compared with 14 (6.1%) in the 2015 to 2016 group. The median peak troponin elevation was 2.2 ug/L in the 2012 to 2013 group and 0.52 ug/L in the 2015 to 2016 group.

**Table 2:** Diagnostic characteristics for 2012 to 2013 and 2015 to 2016.

Variables	All patients (n = 432)	2012–2013 (n = 203)	2015–2016 (n = 229)
Diagnosis, n (%)			
Unstable angina	117 (27)	31 (15)	86 (38)
NSTEMI	229 (53)	117 (58)	112 (49)
STEMI	84 (19)	54 (27)	30 (13)
Mortality Scores, median (IR)			
TIMI Score	3 (2.0)	3 (2.0)	3 (2.0)
GRACE Score	102 (43)	107 (50)	99 (34)
Location on ECG, n (%)			
Anterior	86 (20)	55 (27)	31 (14)
Lateral	107 (25)	64 (32)	43 (19)
Inferior	105 (24)	68 (34)	37 (16)
Posterior	7 (1.6)	6 (3.0)	1 (0.4)
Non-specific	137 (32)	76 (37)	61 (27)
MI Complications, n (%)			
Pulmonary oedema	30 (6.9)	16 (7.9)	14 (6.1)
Shock	7 (1.6)	6 (3.0)	1 (0.4)
High degree block	5 (1.2)	3 (1.5)	2 (0.9)
Peak TnI, ug/L median (IR)	1.3 (5.9)	2.2 (7.5)	0.52 (3.8)
Ejection Fraction %, median (IR)	60(14)	58 (17)	60 (13)

**NSTEMI-** Non-ST segment elevation myocardial infarction

**STEMI-** ST segment elevation myocardial infarction

**TIMI–**Thrombolysis in myocardial infarction

**GRACE–**Global registry of acute coronary events

**ECG**–Electrocardiogram

**MI–**Myocardial infarction

**TnI–**Troponin I

**IR**–Interquartile range

#### Treatment characteristics

Information regarding the treatment characteristics is provided in Table 3. All patients from the 2012 to 2013 cohort were transferred to a tertiary hospital for invasive treatment. One hundred and fifty (75%) were transferred to The Townsville Hospital (TTH), 44 (22%) were transferred to the Mater Hospital Townsville and six (3.0%) were transferred to The Prince Charles Hospital. Referral information for one patient was missing. Only twenty two patients from the 2015 to 2016 cohort were transferred to a tertiary hospital for definitive treatment after first undergoing an inpatient angiogram at MBH. Eighteen (7.9%) patients were transferred to the TTH and four (1.7%) were transferred to the Mater Hospital Townsville.

**Table 3:** Treatment characteristics for 2012 to 2013 and 2015 to 2016.

Variables	All patients (n = 432)	2012–2013 (n = 203)	2015–2016 (n = 229)
Inter-hospital transfers, n (%)	224 (52)	203 (100)	22 (9.1)
Tertiary accepting hospital, n (%)			
The Townsville Hospital	170 (39)	152 (75)	18 (7.9)
Townsville Mater	48 (11)	44 (22)	4 (1.7)
The Prince Charles Hospital	6 (1.4)	6 (3.0)	0
Procedure at accepting hospital, n (%)			
Angiogram	94 (22)	94 (46)	0
Angiogram and PCI	78 (18)	78 (38)	0
Angiogram and CABG	23 (5.3)	23 (11)	0
Rotablation	1 (0.2)	0	1 (0.4)
CABG only	20 (4.6)	0	20 (8.7)
AVR only	1 (0.2)	0	1 (0.4)
Procedure at MBH, n (%)			
Angiogram only	156 (36)	N/A	156 (68)
Angiogram and PCI	73 (17)	N/A	73 (32)
Antiplatelet loading, n (%)			
Clopidogrel loading 300mg	111 (26)	49 (24)	62 (27)
Clopidogrel loading 600mg	139 (32)	97 (48)	42 (18)
Ticagrelor loading 90mg	30 (6.9)	29 (14)	1 (0.4)
Ticagrelor loading 180mg	72 (17)	6 (3.0)	66 (29)
Discharge medications, n (%)			
Aspirin 100mg	342 (79)	152 (75)	190 (83)
Clopidogrel 75mg	163 (38)	89 (44)	74 (32)
Ticagrelor 90mg	77 (18)	27 (13)	50 (22)
ACEI	224 (52)	101 (50)	123 (54)
ARB	55 (13)	21 (10)	34 (15)
Beta blocker	298 (69)	139 (69)	159 (69)
Statin	314 (72.7)	140 (69.0)	174 (76.0)
Nitrate	201 (46.5)	82 (40.4)	119 (52.0)

**N/A–**Not applicable

**PCI–**Percutaneous coronary intervention

**CABG**–Coronary artery bypass graft surgery

**AVR**–Aortic valve replacement

**MBH-** Mackay Base Hospital

**ACEI–**Angiotensin-converting enzyme inhibitor

**ARB**–Angiotensin receptor blocker

In the 2012 to 2013 group 94 (46%) patients received angiography, 78 (38%) received angiography and PCI and 23 (11%) received angiography and CABG at the tertiary accepting hospitals. None of these patients received a procedure at MBH. In the 2015 to 2016 group, one (0.4%) patient received rotablation, 20 (8.7%) patients received a CABG only and one (0.4%) patient received an aortic valve replacement at the tertiary accepting hospital. All patients in the 2015 to 2016 group received either an angiogram or angiogram and PCI at MBH. Ninety seven (48%) patients in the 2012 to 2013 cohort received clopidogrel 600mg loading, compared with 42 (18%) in the 2015 to 2016 cohort. Six (3.0%) patients from the 2012 to 2013 group received ticagrelor 180mg loading, compared with 66 (29%) patients in the 2015 to 2016 group. Patterns of discharge medication prescription were similar between groups.

#### Procedural characteristics

Information regarding the procedural characteristics is provided in Table 4. A radial approach was used in 44 (22%) patients between 2012 and 2013, compared with 196 (86%) patients in the 2015 to 2016 group. Drug eluting stents (DES) were used in 49 (24%) patients between 2012 and 2013 compared with 69 (30%) patients between 2015 and 2016. The left anterior descending was the infarct related artery in 88 (43%) patients in the 2012 to 2013 group and in 85 (37%) patients in the 2015 to 2016 group.

**Table 4:** Procedural characteristics for 2012 to 2013 and 2015 to 2016.

Variables	All patients (n = 432)	2012–2013 (n = 203)	2015–2016 (n = 229)
Approach, n (%)			
Radial	240 (56)	44 (22)	196 (86)
Femoral	179 (41)	143 (70)	36 (16)
Stent Type, n (%)			
Drug eluting stent	118 (27)	49 (24)	69 (30)
Bare metal stent	37 (8.6)	30 (15)	7 (3.1)
Infarct related artery, n (%)			
Left main	17 (3.9)	11 (5.4)	6 (2.6)
Left anterior descending	173 (40)	88 (43)	85 (37)
Circumflex	126 (29)	64 (32)	62 (27)
Right coronary artery	141 (33)	67 (33)	74 (32)
Graft	16 (3.7)	8 (3.9)	8 (3.5)
≥ 3 vessels with ≥ 70% stenosis	74 (17)	50 (25)	24 (11)
No. Vessels treated with PCI, n (%)			
0	253 (59)	104 (51)	149 (65)
1	157 (36)	79 (39)	78 (34)
2	6 (1.4)	4 (2.0)	2 (0.9)

**TIMI–**Thrombolysis in myocardial infarction

#### Time to transfer

Information regarding time to transfer is provided in Table 5. Median time to transfer was one day for patients transferred to a tertiary hospital for an angiogram between 2012 and 2013. No patients were transferred for an angiogram and/or PCI between 2015 and 2016.

**Table 5:** Timing information for 2012 to 2013 and 2015 to 2016.

Variable	2012–2013 (n = 203)	2015–2016 (n = 229)
Time to transfer, days (IR)	1.0 (2.0)	N/A
Time to treatment, days (IR)	5.0 (4.0)	1.8 (2.0)
STEMI	3.0 (2.8)	1.0 (1.9)
NSTEMI	6.0 (4.0)	1.4 (1.9)
Length of stay in tertiary hospital, days (IR)	7.0 (6.0)	N/A
Total length of stay, days (IR)	8.0 (8.0)	4.0 (3.0)

**IR**–Interquartile range

**STEMI**–ST segment elevation myocardial infarction

**NSTEMI**–Non-ST segment elevation myocardial infarction

**N/A**–Not applicable

#### Time to treatment

Information regarding time to treatment is provided in Table 5. Importantly, the median time to treatment dropped from three days between 2012 and 2013 to one day between 2015 and 2016 for patients diagnosed with STEMI. Similarly, for patients diagnosed with NSTEMI the median time to treatment dropped from six days between 2012 and 2013 to 1.4 days between 2015 and 2016.

#### Length of stay

Information regarding length of stay is provided in Table 5. The median length of stay was eight days for patients transferred for invasive treatment between 2012 and 2013 and four days for patients who received in-house catheterisation between 2015 and 2016.

#### Primary outcome

The primary outcome of mortality, recurrent MI or recurrent ischaemia at six months was recorded for 14 (6.9%) patients in the 2012 to 2013 group and for 18 (7.9%) patients in the 2015 to 2016 group (Table 6). Binomial logistic regression analysis of 339 patients, showed no statistically significant difference in the risk of the composite outcome of all-cause mortality, recurrent MI or recurrent ischaemia at six months between patients who were treated between 2012 and 2013 compared with those treated between 2015 and 2016, OR 0.71 (95% CI0.24–2.1), p = 0.54. The model explained between 2.8% (Cox and Snell R square) and 6.5% (Nagelkerke R squared) of the variance in the primary outcome and correctly classified 92% of cases. There was no significant difference between the 2012 to 2013 group and the 2015 to 2016 group in the rate of death (0.5% vs. 0.4%, p = 0.78), recurrent MI (2.5% vs. 3.5%, p = 0.97) and recurrent ischaemia (4.4% vs. 4.4%, p = 0.70). Notably all deaths recorded at six months were secondary to cardiovascular causes. The details of the logistic regression analysis for the primary outcome are detailed in the Supporting Information (S2 Table).

**Table 6:** Primary and secondary clinical outcomes at 1 and 6 months–rates and results of multivariate logistic regression. The data were not adequately powered to performed binomial logistic regression for the individual components of the composite primary and secondary endpoints.

Variables	All patients (n = 432)	2012–2013 (n = 203)	2015–2016 (n = 229)	Odds Ratio (95% CI)	P value
Primary endpoint at 6 months, n (%)	32 (7.4)	14 (6.9)	18 (7.9)	0.71 (0.24–2.1)	0.54
All-cause mortality 6 months, n (%)	2 (0.5)	1 (0.5)	1 (0.4)		
Recurrent MI 6 months, n (%)	13 (3.0)	5 (2.5)	8 (3.5)		
Recurrent ischaemia at 6 months, n (%)	19 (4.4)	9 (4.4)	10 (4.4)		
Secondary endpoint at 1 month, n (%)	7 (1.6)	4 (2.0)	3 (1.3)	1.2 (0.11–13.1)	0.87
All-cause mortality at 1 month, n (%)	0	0	0	N/A	N/A
Recurrent MI at 1 month, n (%)	0	0	0	N/A	N/A
Recurrent ischaemia at 1 month, n (%)	7 (1.6)	4 (2.0)	3 (1.3)	N/A	N/A

**MI**–Myocardial Infarction

**N/A**–Not applicable

#### Secondary outcomes

The secondary outcome of mortality, recurrent MI or recurrent ischaemia at one month was recorded for four (2.0%) patients in the 2012 to 2013 group and for three (1.3%) in the 2015 to 2016 group. This was primarily due to recurrent ischaemia at one month in both groups, as there were no cases of mortality or recurrent MI at one month in either group (Table 6). Binomial logistic regression of 341 patients showed no statistically significant difference in the risk of the secondary outcome at one month between patients treated in 2012 and 2013 compared with 2015 and 2016, OR 1.2 (95% CI0.11–13.1), p = 0.87 (Table 6). The model explained between 3.5% (Cox and Snell R square) and 22% (Nagelkerke R squared) of the variance in the secondary outcome and correctly classified 98% of cases. The details of the logistic regression analysis for the secondary outcome are detailed in the Supporting Information (S3 Table). Binomial logistic regression analyses were not performed for individual components of the composite primary and secondary outcomes because the data were not adequately powered to do so.

There were no cases for major TIMI bleeding at one and six months for either group.

### Patient satisfaction surveys

Patient satisfaction surveys were mailed to 419 patients. We received completed surveys from 159 respondents. Two returned surveys were excluded because information was ambiguous (multiple answers provided for each question) and another was incomplete. We included 156 eligible returned surveys for data analysis.

#### Participant characteristics

The participant characteristics from the patient satisfaction surveys are detailed in the Supporting Information (S4 Table) We received 53 completed surveys from patients treated between 2012 and 2013 and 103 completed surveys from patients treated between 2015 and 2016. Patients aged 60 to 69 years comprised 36% of respondents (45% of patients in this age bracket in the 2012 to 2013 group and 31% in the 2015–2016 group). Males made up 65% of all respondents with 68% in the 2012 to 2013 group and 64% in the 2015 to 2016 group.

#### Summated score analysis

The Cronbach’s alpha coefficient for the scale increased from 0.75 to 0.81 when the item ‘satisfaction with the mode of transport’ was removed. Therefore this item was excluded from the summated score analysis. Additionally as none of the respondents from 2015 to 2016 were transferred for treatment this item could not be compared between groups. The summated patient satisfaction score could range from a low of two to a high of ten.

**Fig 2:**
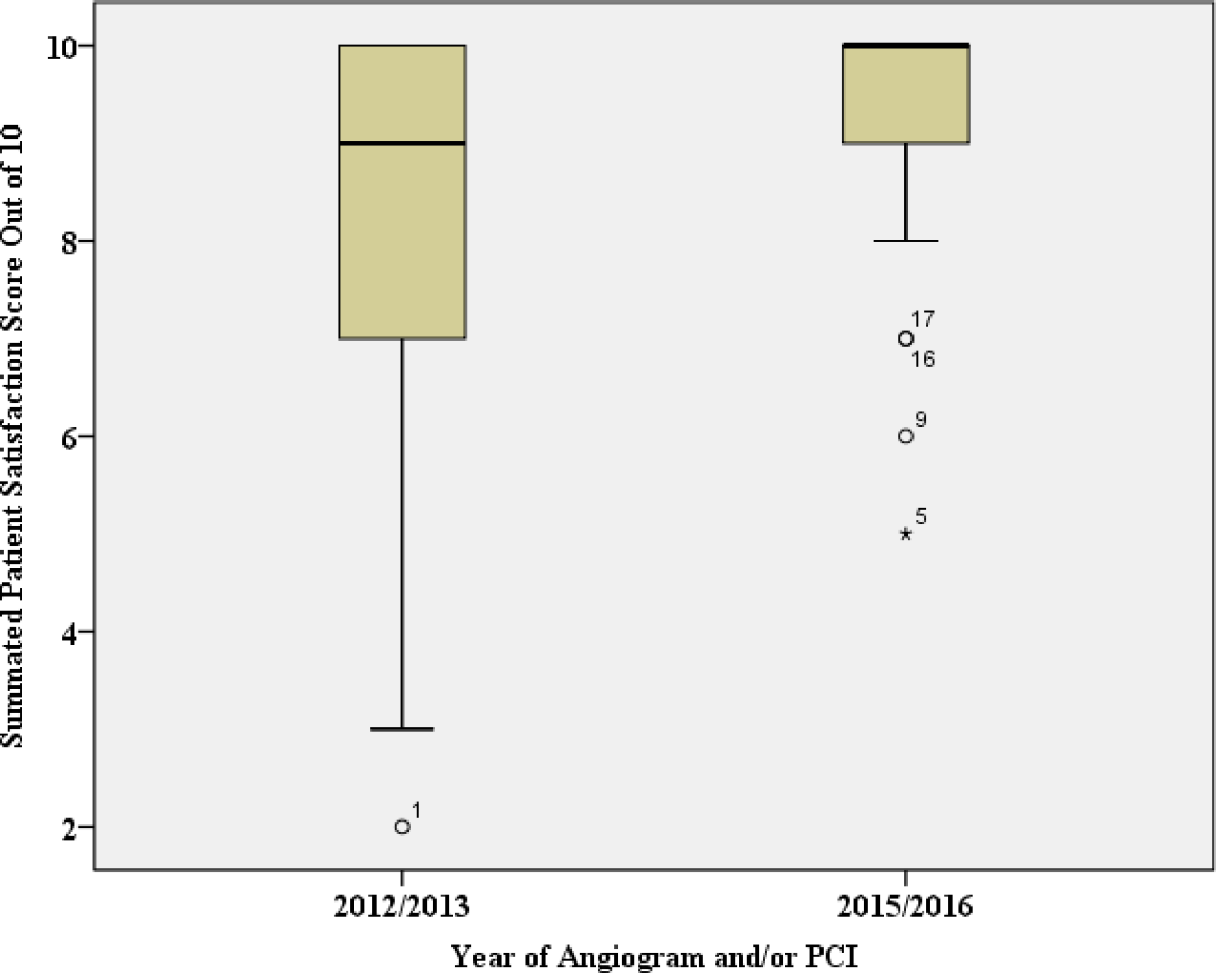
Box plots comparing median summated patient satisfaction scores between 2012–2013 and 2015–2016.

The median summated patient satisfaction score was nine for patients treated from 2012 to 2013, compared to a median of ten for patients treated from 2015 to 2016 (Fig 2). The Mann-Whitney U test indicated that the summated patient satisfaction score was statistically significantly greater for patients admitted between 2015 and 2016 compared with patients admitted between 2012 and 2013, U = 1918, p <0.05 (two tailed). The sum of ranks was higher for the 2015 to 2016 group (8897) compared with the 2012 to 2013 group (3350).

#### Single item analysis

Details of the single item analysis are provided in the Supporting Information (S5 Table and S4 Appendix).

#### Thematic analysis

Details of the thematic analysis are provided in the Supporting Information (S6 Table and S5 Appendix).

### Estimated cost savings

#### Inter-hospital transfer

From 2015 to 2016 only 22 patients were transferred for definitive treatment, therefore 207 patients avoided inter-hospital transfer and were treated locally. The estimated cost saving per patient is shown in Table 7. A further commentary is provided in the Supporting Information (S6 Appendix).

**Table 7:** Estimated cost savings generated from providing intervention for 207 patients in MBH from February 2015 to January 2016.

Description of expenses prevented	Calculation of cost	Total
Royal Flying Doctor Service travel	207 x $3060	$633 420
Queensland Ambulance Service travel	207 x 2 x $832	$344 468
Escort travel and patient return by car	207 x $233	$48 351
Escort accommodation	207 x 3 x $60	$37 260
Bed- day cost MBH CCU	207 x 3.2 x $2 336	$1 934 208
**Total savings for the study period**		$2 997 707

**MBH–**Mackay Base hospital

**CCU**- Coronary Care Unit

#### Bed day cost

The estimated bed-day cost for one MBH CCU bed was quoted at $2336 per night. By reducing the median length of stay by four days in the 2015 to 2016 group, we calculated an estimated cost saving of $1.9 million per annum for the 207 patients who were treated regionally.

#### Total cost saving

It was estimated that avoiding inter-hospital transfer for 207 patients in 2015 to 2016 and reducing the median length of stay for these patients amounted to a cost saving of approximately $2.9 million per annum.

$633420+$344468+48351+$37260+$1.9million=$2.9million

This translated to a cost saving of $14 481 per patient.

$2.9million/207=$14481

#### Expense 2015–2016

The average cost for inpatient care from 2015 to 2016 was estimated at $13 555 per patient.

#### Estimated cost saving percentage

Potential cost per patient from 2015 to 2016 with inpatient care and inter hospital transfer was calculated at $28 036.

$13555+$14481=$28036

Therefore we estimated a proportional cost saving of 51% per patient treated regionally from 2015 to 2016.

$14481/28036x100=51%

## Discussion

Our study strongly supports the provision of regional in-house angiography and/or PCI for the management of ACS patients in MBH. The present study shows that in-house angiography and/or PCI minimises delays to treatment by 3.2 days, reduces the median length of stay by four days, significantly improves patient satisfaction and reduces proportional treatment costs by $14 481 (51%) per patient. Currently, however it appears that the introduction of a CCL for the management of ACS does not translate to a further reduction in the risk of mortality, recurrent MI and recurrent ischaemia at one and six months. The findings of our study have considerable clinical and economic implications for the regional hospitals, tertiary referral hospitals and the area health networks.

While it is possible that in-house angiography and/or PCI may improve health outcomes, our study demonstrated similar risk in the composite of mortality, recurrent MI or recurrent ischaemia at one and six months between patients receiving in-house angiography and inter hospital transfer. These results are supported by previous trials and meta-analyses which show that earlier PCI does not lead to reduced rates of mortality and recurrent MI at one and six amongst high risk NSTEACS patients receiving an early invasive strategy.[16–22] Results from these trials however may not be directly applicable to our work as the median time to angiography and/or PCI in the early arm of these studies was frequently shorter than what was achieved amongst the in-house angiography group in our study. For example, the median time to angiography in the early group was 14 hours in the TIMACS trial, whereas the median time to treatment was 1.8 days amongst patients who received in-house catheterisation in our study.[16] Furthermore the incidence of the outcomes in our study was low, so while our results suggest there is no further reduction in major adverse cardiac events (MACE) with the introduction of a local interventional service, our study is not powered enough to rule out a small true effect.

It is likely that in-house angiography and/or PCI improve mortality rates over the longer term. However in our study this endpoint was analysed for the short- and intermediate- term and long-term extrapolation is not automatically possible. This is supported by previous literature, such as the RITA trial. This trial failed to demonstrate a difference in mortality after one year, but demonstrated a significant reduction in mortality amongst patients treated with a routine invasive strategy after five years follow up.[6, 23] Therefore further long-term analyses of the patients included in this study are most likely required to establish a difference in mortality between groups.

Additionally we were unable to demonstrate a significant difference in the rate of recurrent MI due to our high threshold for diagnosing this endpoint. We required that patients exhibit clinical symptoms consistent with ischaemia and biomarker evidence with/without appropriate ECG changes as per Australian guidelines.[3] This is unlike previous studies which included isolated biomarker elevations in their recurrent MI endpoint and primary outcome.[24] ^[^^18^^]^ Indeed the majority of events contributing to the MI endpoint in these trials were minor biomarker elevations.[24] A report from the Mayo Group suggests that isolated minor biomarker elevations post PCI do not convey significant short or longer term risk therefore these events were not included in our recurrent MI endpoint.[25] Previous trials which recorded higher rates of recurrent MI infrequently included ischaemic symptoms as recommended in the universal definition for periprocedural MI.[26] These trials also utilised lower biomarker elevations that what was suggested by the Society of Cardiovascular Angiography and Interventions (SCAI) definition.[27] The prognostic significance of biomarker elevations post-PCI is unclear making it difficult for trials to distinguish between periprocedural and spontaneous MIs.[26] Therefore many trials which recorded higher rates of recurrent MI included high proportions of periprocedural events.[24, 28] We did not include periprocedural events in our definition of recurrent MI, possibly further explaining our low rate of recurrent MI amongst groups.

Furthermore, we may not have detected a possible clinical benefit from the implementation of a regional catheterisation laboratory because at the time this study was conducted the catheterisation laboratory at MBH was only operating during working hours three days a week. An emergency 24/7 catheterisation service was not available and therefore there was a subset of ACS patients who may have been directly transferred from the ED to tertiary hospitals for catheterisation between 2015 and 2016. Analysing this data was beyond the scope of the current study. A regional catheterisation laboratory which provides an emergency 24/7 service may demonstrate improved clinical outcomes. Therefore this study should be repeated when the MBH catheterisation laboratory offers 24/7 emergency angiography and/or PCI.

We did not record any events of major TIMI bleeding in either group. Therefore it is unclear whether earlier regional angiography and/or PCI provides an additional safety benefit. Recent literature indicates that radial access compared with femoral access minimises major non-CABG related bleeding events.[29] Our research showed a shift to a predominant radial approach in MBH between 2015 and 2016 compared with 2012 and 2013 (86% vs. 16%). Statewide 45% of PCI cases performed during 2015 were via a radial approach.[10] Certainly, previous reports of major bleeding events are low amongst patients receiving early and delayed intervention (0.5% vs. 1.0%).[28] Therefore whilst the adoption of a predominant radial approach may explain the lack of bleeding events, we were most likely underpowered to detect a difference in this endpoint.

Our study demonstrated a significant reduction in the median time to treatment amongst patients receiving in-house angiography and/or PCI. Our practice now more closely mirrors what is recommended by Australian guidelines. These suggest that high risk NSTEACS patients should receive PCI within 48 hours of their diagnosis.[3] In 2015–2016 the median time to angiography and/or PCI for NSTEMI patients dropped to 1.8 days indicating that at least 50% of patients with this diagnosis who presented to our facility were treated within the recommended time frame. The optimal timing for invasive management of high risk NSTEACS patients is still under contention however a recently published meta-analysis suggests that stabilised high risk NSTEACS should receive early intervention within 24 hours.[30] Another analysis suggests that the optimal time to intervention for these patients is between 20 and 40 hours. Therefore whilst the delay associated with inter-hospital transfer currently appears safe in the short term, further reductions in time to treatment for high risk NSTEACS patients appear to be warranted.[31] Despite the provision of in-house catheterisation we were unable to offer a unanimously early approach (within 24 hours) to all high risk NSTEACS patients. This is expected to improve as the catheterisation laboratory develops into an emergency 24/7 service.

The median time to treatment for STEMI patients dropped from three days to one day. While this indicates an improvement in time to treatment for regional STEMI patients, it does not meet Australian guidelines. Patients diagnosed with ‘late presentation STEMI’ and ‘aborted STEMI’ accounted for the majority of those who were diagnosed with STEMI between 2015 and 2016 and received intervention after one day. Australian guidelines for these subsets of STEMI patients recommend primary PCI upon presentation. The reasons for delay to treatment include the limited operating hours for the catheterisation laboratory (3.5 days per week) and resource limitations with after-hours PCI services currently unavailable. Ultimately the long-term aim for the MBH catheterisation laboratory is to run a 24/7 service and thereby meet Australian guidelines for all STEMI patients, including those presenting late and with aborted STEMIs. We also aim to meet the time recommendations for all thrombolysed STEMI patients who present to MBH for routine PCI from smaller rural hospitals. [13]

Our data indicate that prior to the establishment of the regional catheterisation laboratory in MBH, the major delay in time to treatment potentially occurred after patients were admitted to the accepting hospital. The median time to transfer was recorded as one day between 2012 and 2013 however after minimising the need for inter-hospital transfer the drop in median time to treatment was three fold (3.2 day reduction) for patients treated from 2015 to 2016. The delay after admission to the accepting hospital is likely multi-factorial. Further research is required to elicit the exact causes for this delay.

Furthermore we also showed that patent satisfaction is significantly improved with the provision of regional in-house catheterisation. This outcome measure is increasingly viewed as an important indicator of quality service provision, yet literature regarding patient satisfaction is scarce. This is especially true for the acute setting. Previous data indicate that a proportion of patients prefer to be treated at their local hospital, despite acknowledging that transfer for PCI provides optimal acute care.[32] Past literature also shows that rural physicians tend to overestimate the patient-perceived value of nonmedical aspects of care, such as the importance of proximity to home, and underestimate the importance of being cared for in a comprehensive medical centre.[33] Therefore the provision of in-house catheterisation in Mackay represents a unique innovation that simultaneously maximises patient satisfaction whilst improving access to high-level medical care.

We hypothesised that patient satisfaction would be increased amongst patients receiving in-house angiography and/or PCI due to the elimination of inter hospital transfer. Only 22 ACS patients who underwent angiography in Mackay between 2015 and 2016 required further inter-hospital transfer for cardiothoracic services currently unavailable at MBH. Our assumption is supported by previous studies which identify financial challenges and the displacement from family as deterrents to inter hospital transfer.[32] Indeed our results support our hypothesis. Thematic analysis of the comments showed that patients were predominantly satisfied with their interaction with hospital staff and the service provision at the local and tertiary accepting hospitals but were dissatisfied with the extended waiting periods for transfer and treatment. Interestingly, quantitative and thematic analysis showed that patients were very satisfied with mode of transport to the accepting hospital, but expressed dissatisfaction with the lack of a dedicated transport service for patient return. Recent data suggests that patients who undergo elective PCI demonstrate comparable satisfaction between early discharge and overnight observation.[34] This suggests that in contrast to minimising delays to treatment, shortening the duration of hospitalisation after intervention may have less influence on patient satisfaction.

Previous data indicates that careful explanation and education is the hallmark of patient satisfaction in the acute setting.[32] In our study the theme of patient education was greater amongst patients receiving in-house angiography and may have alleviated potential concerns and thereby increased satisfaction. The treatment of ACS however represents a chaotic and stressful event where patient education continues to be a challenge as identified by the American Heart Association.[35]

The establishment of the regional catheterisation laboratory in Mackay represented a demand for increased capital investment in the North Queensland region. The provision of in-house catheterisation reduced the proportional cost per patient and thereby liberated additional funds for allocation into other patient services. Moreover the reduction in the number of bed-days before treatment represented an increase in the capacity of the hospital to accommodate a larger quota of patients each year. This reduction yielded cost savings as it translated to a 50% reduction in the median length of stay, from eight days to four days, for patients treated between 2015 and 2016. Furthermore redirecting catheterisation from tertiary hospitals to our regional hospital signifies a positive shift in the standard of care provided by MBH, which now delivers a more robust medical service for the management of ACS. Equally important, the improved patient flow and reduced bed pressure at the tertiary hospitals likely correspond to additional cost savings to the health network. Patients were principally transferred to TTH between 2013 and 2013. Therefore the establishment of the CCL in MBH may be expected to especially improve patient flow management in this tertiary centre.

Previous data examining cost implications support our findings. Several studies suggest that cost savings are to be made when a hospital provides earlier and local intervention to ACS patients.[34, 36] Data regarding the treatment of NSTEACS indicates that a routinely invasive strategy is likely to be considered cost effective compared to a conservative strategy.[37] The RITA trial however indicates that this benefit may be restricted to high and intermediate risk patients.[38] Further data also demonstrate that an early invasive strategy is likely to be less costly compared with a delayed invasive strategy in NSTEACS patients.[39] Relevantly, recent literature from the North Queensland region shows that a shift towards local service delivery yields net savings mainly due to the avoidance of travel costs. [40]

When estimating cost savings we excluded the cost of establishing and maintaining the catheterisation laboratory. This is because the ACS patients represented a subset of the total patients serviced by the regional catheterisation laboratory. For example from 2015 to 2016 less than a third of the total patients treated in the CCL were diagnosed with ACS. To perform an incremental cost effective analysis of establishing the regional catheterisation laboratory in Mackay the cost savings for all patients treated by the service would have to be accounted for. Additionally the incremental costs per prevented cardiac event should be estimated for patients treated between 2012 and 2013 against those treated between 2015 and 2016. This was beyond the scope of our study.

The data in our study also provides, for the first time, a cross-sectional description of the diagnostic, treatment and procedural characteristics of ACS patients receiving intervention in MBH. We did not include STEMI patients who were directly transferred from the Emergency Department to tertiary centres for PCI, possibly accounting for the smaller percentage of STEMI patients recorded from 2015 to 2016. Our data indicated that a higher proportion of patients received conservative treatment (or angiography only) between 2015 and 2016. This probably reflected the practices of a newly established catheterisation laboratory without access to onsite cardiothoracic surgery. Indeed an annual report from 2015 showed that, unlike the seven other CCLs operating in Queensland, the majority of PCI cases in Mackay occurred in the elective setting.[10] Our data also showed a trend towards ticagrelor loading between 2015 and 2016. This appeared justified by results from the Study of Platelet Inhibition and Patient Outcomes trial which showed that ticagrelor significantly reduced the rate of death from vascular causes, MI or stroke amongst ACS patients compared with clopidogrel.[41] Additionally, DES were more commonly used between 2015 and 2016. In fact recent reports indicate that the use of DES was highest in MBH, compared with the other CCLs operating in Queensland. Most cases demanded the deployment of one stent, indeed this is congruent with previous data showing across all centres in Queensland, 1.5 stents on average were used per PCI case.[10]

### Strengths

To our knowledge we have conducted the first single-centre study in Australia comparing the model of inter hospital transfer with regional angiography and/or PCI in terms of time to invasive treatment, length of hospital stay, clinical outcomes, patient satisfaction and cost savings for the entire spectrum of ACS. Our study provides new insights into the management of ACS in the regional setting. Ultimately, the results from this study show that the provision of a regional catheterisation laboratory reduces delays to treatment, minimises hospital stays, improves patient satisfaction and minimises cost. Thereby our work provides the safety and confidence to offer interventional cardiac services regionally.

### Limitations

The study had several limitations. First, retrospective data were used to analyse clinical outcomes between groups therefore this data was subject to confounding bias. To mitigate this we employed multivariate logistic regression; although residual confounding bias could not be excluded. Second, this was a single centre study therefore our results may not be directly applicable to other centres nationally and internationally. Third, selection bias could not be excluded. To address selection bias we used a consecutive sample of all ACS patients presenting to MBH CCU in both groups. Fourth, the included sample size may be underpowered to prove a small true difference in the composite clinical endpoints between groups. Fifth, we did not use a previously validated patient satisfaction survey. To maximise validity we pilot tested the survey and adjusted it repeatedly prior to distributing the final version. To ensure reliability of our scale we used the Cronbach’s alpha coefficient to measure internal consistency. Lastly, the recurrent ischaemia endpoint is partially subjective therefore we advise caution when interpreting this result. Further research would be required to analyse this endpoint separately.

## Conclusion

This study suggests that the provision of in-house angiography and/or PCI represents a unique innovation that simultaneously minimises delays to treatment by 3.2 days, reduces the median length of stay by four days, significantly improves patient satisfaction and reduces proportional treatment costs by $14 481 (51%) per patient. Currently, from this study, it appears that in-house catheterisation does not further reduce the risk of mortality, recurrent MI and recurrent ischaemia at one and six months.

## Supporting information

S1 Appendix Definition for major non-CABG related TIMI bleeding. (DOCX)

S2 Appendix Interview pro-forma. (DOCX)

S3 Appendix A: Cardiology satisfaction survey- patient information leaflet. B: Cardiology satisfaction survey.
(DOCX)

S4 Appendix Single item analysis from patient satisfaction survey. (DOCX)

S5 Appendix Thematic analysis from patient satisfaction survey. (DOCX)

S6 Appendix Estimated cost savings from inter-hospital transfer. (DOCX)

S1 Fig Box plots comparing median patient satisfaction scores for overall convenience between 2012–2013 and 2015–2016. (DOCX)

S2 Fig Box plots comparing median patient satisfaction scores for length of time spent waiting between 2012–2013 and 2015–2016. (DOCX)

S3 Fig Box plot showing the median patient satisfaction score for mode of transport between 2012 and 2013. (DOCX)

S1 Table A: TIMI scoring for unstable angina/NSTEMI. B: TIMI scoring for STEMI. C: GRACE scoring for ACS.
(DOCX)

S2 Table Multivariate logistic regression for the primary outcome—composite of all-cause mortality, recurrent MI and recurrent ischaemia at 6 months. **PCI–**Percutaneous coronary intervention. **CABG–**Coronary artery bypass graft surgery. **GRACE–**Global registry of acute coronary events. **TIMI**- Thrombolysis in myocardial infarction.
(DOCX)

S3 Table Multivariate logistic regression for the secondary outcome—composite of all-cause mortality, recurrent MI and recurrent ischaemia at 1 month. **PCI–**Percutaneous coronary intervention. **CABG–**Coronary artery bypass graft surgery. **GRACE–**Global registry of acute coronary events. **TIMI**- Thrombolysis in myocardial infarction.
(DOCX)

S4 Table Patient characteristics from satisfaction surveys. **PCI**–Percutaneous coronary intervention.
(DOCX)

S5 Table Summated score analysis and individual score analysis of patient satisfaction surveys. **IR**–Interquartile range. **N/A**–Not applicable.
(DOCX)

S6 Table Illustrative quotations supporting thematic analysis of patient satisfaction surveys. (DOCX)

## References

[1] Murray CJ, Lopez AD. Alternative projections of mortality and disability by cause 1990–2020: Global Burden of Disease Study. Lancet. 1997;349(9064):1498–504. Epub 1997/05/24. 10.1016/S0140-6736(96)07492-2 . 9167458

[2] Hamm CW, Bassand JP, Agewall S, Bax J, Boersma E, Bueno H, et al ESC Guidelines for the management of acute coronary syndromes in patients presenting without persistent ST-segment elevation: The Task Force for the management of acute coronary syndromes (ACS) in patients presenting without persistent ST-segment elevation of the European Society of Cardiology (ESC). Eur Heart J. 2011;32(23):2999–3054. Epub 2011/08/30. 10.1093/eurheartj/ehr236 . 21873419

[3] Chew DP, Scott IA, Cullen L, French JK, Briffa TG, Tideman PA, et al National Heart Foundation of Australia and Cardiac Society of Australia and New Zealand: Australian clinical guidelines for the management of acute coronary syndromes 2016. Med J Aust. 2016;205(3):128–33. Epub 2016/07/29. . 27465769 10.5694/mja16.00368

[4] Andersen HR, Nielsen TT, Rasmussen K, Thuesen L, Kelbaek H, Thayssen P, et al A comparison of coronary angioplasty with fibrinolytic therapy in acute myocardial infarction. N Engl J Med. 2003;349(8):733–42. Epub 2003/08/22. 10.1056/NEJMoa025142 . 12930925

[5] Wallentin L, Lagerqvist B, Husted S, Kontny F, Stahle E, Swahn E. Outcome at 1 year after an invasive compared with a non-invasive strategy in unstable coronary-artery disease: the FRISC II invasive randomised trial. FRISC II Investigators. Fast Revascularisation during Instability in Coronary artery disease. Lancet. 2000;356(9223):9–16. Epub 2000/07/13. . 10892758 10.1016/s0140-6736(00)02427-2

[6] Fox KA, Poole-Wilson P, Clayton TC, Henderson RA, Shaw TR, Wheatley DJ, et al 5-year outcome of an interventional strategy in non-ST-elevation acute coronary syndrome: the British Heart Foundation RITA 3 randomised trial. Lancet. 2005;366(9489):914–20. Epub 2005/09/13. 10.1016/S0140-6736(05)67222-4 . 16154018

[7] Cannon CP, Weintraub WS, Demopoulos LA, Vicari R, Frey MJ, Lakkis N, et al Comparison of early invasive and conservative strategies in patients with unstable coronary syndromes treated with the glycoprotein IIb/IIIa inhibitor tirofiban. N Engl J Med. 2001;344(25):1879–87. Epub 2001/06/23. 10.1056/NEJM200106213442501 . 11419424

[8] Welfare AIoHa. Cardiovascular medicines and primary heatlh care: a regional analysis Canberra Australian Government, 2010.

[9] Fox KA, Goodman SG, Anderson FA Jr., Granger CB, Moscucci M, Flather MD, et al From guidelines to clinical practice: the impact of hospital and geographical characteristics on temporal trends in the management of acute coronary syndromes. The Global Registry of Acute Coronary Events (GRACE). Eur Heart J. 2003;24(15):1414–24. Epub 2003/08/12. . 12909070 10.1016/s0195-668x(03)00315-4

[10] Starmer G HC, Lim R et al Queensland Cardiac Outcomes Registry (QCOR) Interventional Cardiology 2015 Annual Report. Queensland: Queensland Government. Department of Health, 2015.

[11] Mehran R, Rao SV, Bhatt DL, Gibson CM, Caixeta A, Eikelboom J, et al Standardized bleeding definitions for cardiovascular clinical trials: a consensus report from the Bleeding Academic Research Consortium. Circulation. 2011;123(23):2736–47. Epub 2011/06/15. 10.1161/CIRCULATIONAHA.110.009449 . 21670242

[12] Corporation R. Patient satisfaction questionnaire from RAND Health California, USA2016 [cited 2016].

[13] Cantor WJ, Fitchett D, Borgundvaag B, Ducas J, Heffernan M, Cohen EA, et al Routine Early Angioplasty after Fibrinolysis for Acute Myocardial Infarction. New England Journal of Medicine. 2009;360(26):2705–18. 10.1056/NEJMoa0808276 . 19553646

[14] McNamee R. Regression modelling and other methods to control confounding. Occup Environ Med. 2005;62(7):500–6, 472. Epub 2005/06/18. 10.1136/oem.2002.001115 15961628 PMC1741049

[15] Gliem JA GR. Calculating, interpreting and reporting cronbach's alpha reliability coefficient for Likert-type scales. [Midwest Research to Practice Conference in Adult, Continuing, and Community Education]. In press 2003.

[16] Mehta SR, Granger CB, Boden WE, Steg PG, Bassand JP, Faxon DP, et al Early versus delayed invasive intervention in acute coronary syndromes. N Engl J Med. 2009;360(21):2165–75. Epub 2009/05/22. 10.1056/NEJMoa0807986 . 19458363

[17] Badings EA, The SH, Dambrink JH, van Wijngaarden J, Tjeerdsma G, Rasoul S, et al Early or late intervention in high-risk non-ST-elevation acute coronary syndromes: results of the ELISA-3 trial. EuroIntervention. 2013;9(1):54–61. 10.4244/EIJV9I1A9. . 23685295

[18] Montalescot G, Cayla G, Collet JP, Elhadad S, Beygui F, Le Breton H, et al Immediate vs delayed intervention for acute coronary syndromes: a randomized clinical trial. Jama. 2009;302(9):947–54. 10.1001/jama.2009.1267. . 19724041

[19] Navarese EP, De Servi S, Gibson CM, Buffon A, Castriota F, Kubica J, et al Early vs. delayed invasive strategy in patients with acute coronary syndromes without ST-segment elevation: a meta-analysis of randomized studies. QJM. 2011;104(3):193–200. Epub 2011/01/26. 10.1093/qjmed/hcq258 . 21262739

[20] Rajpurohit N, Garg N, Garg R, Choudhary A, Fresen J, Boren S, et al Early versus delayed percutaneous coronary intervention for patients with non-ST segment elevation acute coronary syndrome: a meta-analysis of randomized controlled clinical trials. Catheter Cardiovasc Interv. 2013;81(2):223–31. Epub 2012/04/11. 10.1002/ccd.24439 . 22488783

[21] Zhang S, Ge J, Yao K, Qian J. Meta-analysis of early versus deferred revascularization for non-ST-segment elevation acute coronary syndrome. Am J Cardiol. 2011;108(9):1207–13. Epub 2011/08/30. 10.1016/j.amjcard.2011.06.035 . 21872193

[22] Milasinovic D, Milosevic A, Marinkovic J, Vukcevic V, Ristic A, Asanin M, et al Timing of invasive strategy in NSTE-ACS patients and effect on clinical outcomes: A systematic review and meta-analysis of randomized controlled trials. Atherosclerosis. 2015;241(1):48–54. 10.1016/j.atherosclerosis.2015.04.808 25966439

[23] Fox KA, Poole-Wilson PA, Henderson RA, Clayton TC, Chamberlain DA, Shaw TR, et al Interventional versus conservative treatment for patients with unstable angina or non-ST-elevation myocardial infarction: the British Heart Foundation RITA 3 randomised trial. Randomized Intervention Trial of unstable Angina. Lancet. 2002;360(9335):743–51. Epub 2002/09/21. . 12241831 10.1016/s0140-6736(02)09894-x

[24] Riezebos RK, Ronner E, Ter Bals E, Slagboom T, Smits PC, ten Berg JM, et al Immediate versus deferred coronary angioplasty in non-ST-segment elevation acute coronary syndromes. Heart. 2009;95(10):807–12. 10.1136/hrt.2008.154815. . 19098058

[25] Prasad A, Rihal CS, Lennon RJ, Singh M, Jaffe AS, Holmes DR Jr. Significance of periprocedural myonecrosis on outcomes after percutaneous coronary intervention: an analysis of preintervention and postintervention troponin T levels in 5487 patients. Circ Cardiovasc Interv. 2008;1(1):10–9. Epub 2008/08/01. 10.1161/CIRCINTERVENTIONS.108.765610 . 20031650

[26] Thygesen K, Alpert JS, Jaffe AS, Simoons ML, Chaitman BR, White HD, et al Third universal definition of myocardial infarction. J Am Coll Cardiol. 2012;60(16):1581–98. Epub 2012/09/11. 10.1016/j.jacc.2012.08.001 . 22958960

[27] White H. Avatar of the universal definition of periprocedural myocardial infarction. J Am Coll Cardiol. 2013;62(17):1571–4. Epub 2013/10/19. 10.1016/j.jacc.2013.08.721 . 24135582

[28] Thiele H, Rach J, Klein N, Pfeiffer D, Hartmann A, Hambrecht R, et al Optimal timing of invasive angiography in stable non-ST-elevation myocardial infarction: the Leipzig Immediate versus early and late PercutaneouS coronary Intervention triAl in NSTEMI (LIPSIA-NSTEMI Trial). Eur Heart J. 2012;33(16):2035–43. Epub 2011/11/24. 10.1093/eurheartj/ehr418 . 22108830

[29] Valgimigli M, Gagnor A, Calabro P, Frigoli E, Leonardi S, Zaro T, et al Radial versus femoral access in patients with acute coronary syndromes undergoing invasive management: a randomised multicentre trial. Lancet. 2015;385(9986):2465–76. Epub 2015/03/21. 10.1016/S0140-6736(15)60292-6 . 25791214

[30] Jiang M, Mao JL, Pu J, He B. Timing of early angiography in non-ST elevation acute coronary syndrome. J Invasive Cardiol. 2014;26(2):47–54. Epub 2014/02/04. . 24486660

[31] Mahmoud KD, Hillege HL, Lennon RJ, Gersh BJ, Holmes DR Jr. Timing of intervention and outcome in non-ST-elevation acute coronary syndromes: there is risk on both sides of the curve. Int J Cardiol. 2014;177(1):23–4. Epub 2014/12/17. 10.1016/j.ijcard.2014.09.057 . 25499327

[32] Henry JT, Christiansen E, Garberich RF, Handran CB, Larson DM, Unger BT, et al Satisfaction with emergent transfer for percutaneous coronary interventions on patients with ST-segment-elevation myocardial infarction and their families. Circ Cardiovasc Qual Outcomes. 2014;7(2):244–50. Epub 2014/03/07. 10.1161/CIRCOUTCOMES.113.000641 . 24594547

[33] Mohr NM, Wong TS, Faine B, Schlichting A, Noack J, Ahmed A. Discordance Between Patient and Clinician Experiences and Priorities in Rural Interhospital Transfer: A Mixed Methods Study. J Rural Health. 2016;32(1):25–34. Epub 2015/07/16. 10.1111/jrh.12125 . 26174410

[34] Glaser R, Gertz Z, Matthai WH, Wilensky RL, Weiner M, Kolansky D, et al Patient satisfaction is comparable to early discharge versus overnight observation after elective percutaneous coronary intervention. J Invasive Cardiol. 2009;21(9):464–7. Epub 2009/09/04. . 19726820

[35] Jacobs AK, Antman EM, Faxon DP, Gregory T, Solis P. Development of systems of care for ST-elevation myocardial infarction patients: executive summary. Circulation. 2007;116(2):217–30. Epub 2007/06/01. 10.1161/CIRCULATIONAHA.107.184043 . 17538045

[36] Lamy A, Tong WR, Bainey K, Gafni A, Rao-Melacini P, Mehta SR. Cost implication of an early invasive strategy on weekdays and weekends in patients with acute coronary syndromes. Can J Cardiol. 2015;31(3):314–9. Epub 2015/03/10. 10.1016/j.cjca.2014.11.025 . 25746022

[37] Dijksman LM, Hirsch A, Windhausen F, Asselman FF, Tijssen JG, Dijkgraaf MG, et al Cost-effectiveness of early versus selectively invasive strategy in patients with acute coronary syndromes without ST-segment elevation. Int J Cardiol. 2009;131(2):204–11. Epub 2008/01/18. 10.1016/j.ijcard.2007.10.019 . 18199496

[38] Henriksson M, Epstein DM, Palmer SJ, Sculpher MJ, Clayton TC, Pocock SJ, et al The cost-effectiveness of an early interventional strategy in non-ST-elevation acute coronary syndrome based on the RITA 3 trial. Heart. 2008;94(6):717–23. 10.1136/hrt.2007.127340 . 18032459

[39] Bainey KR, Gafni A, Rao-Melacini P, Tong W, Steg PG, Faxon DP, et al The cost implications of an early versus delayed invasive strategy in Acute Coronary Syndromes: the TIMACS study. J Med Econ. 2014;17(6):415–22. Epub 2014/04/08. 10.3111/13696998.2014.911184 . 24702256

[40] Thaker DA, Monypenny R, Olver I, Sabesan S. Cost savings from a telemedicine model of care in northern Queensland, Australia. Med J Aust. 2013;199(6):414–7. Epub 2013/09/17. . 24033216 10.5694/mja12.11781

[41] Wallentin L, Becker RC, Budaj A, Cannon CP, Emanuelsson H, Held C, et al Ticagrelor versus clopidogrel in patients with acute coronary syndromes. N Engl J Med. 2009;361(11):1045–57. Epub 2009/09/01. 10.1056/NEJMoa0904327 . 19717846

