# Supplementary material for: Regional to tertiary inter-hospital transfer versus in-house percutaneous coronary intervention in acute coronary syndrome
Source: PLoS One. 2018 Jun 21;13(6):e0198272. doi: 10.1371/journal.pone.0198272 (PMC6013182; doi:10.1371/journal.pone.0198272)
Supplement: S1 Appendix — (DOCX) [file pone.0198272.s001.docx]

**S1 Appendix. Definition for major non-CABG related TIMI bleeding**

- Any intracranial bleeding (excluding micro-haemorrhages <10 mm evident only on gradient-echo MRI)
- Clinically overt signs of haemorrhage associated with a drop in haemoglobin of ≥5 g/dL or a ≥15% absolute decrease in haematocrit
- Fatal bleeding (bleeding that directly results in death within 7 days)
