# Supplementary material for: Regional to tertiary inter-hospital transfer versus in-house percutaneous coronary intervention in acute coronary syndrome
Source: PLoS One. 2018 Jun 21;13(6):e0198272. doi: 10.1371/journal.pone.0198272 (PMC6013182; doi:10.1371/journal.pone.0198272)
Supplement: S2 Appendix — (DOCX) [file pone.0198272.s002.docx]

**S2 Appendix. Interview Pro-forma**


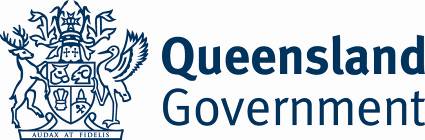


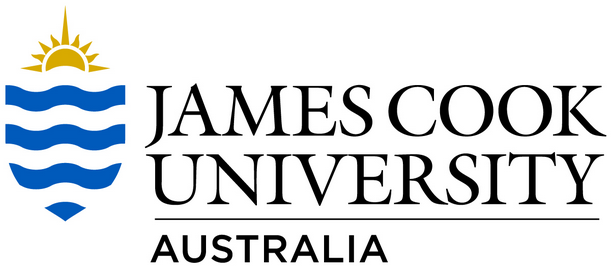


**Interview Pro-forma**

**Patient name __________________________________________________**

**Patient D.O.B _________________________________________________**

**Introduction (first 2 minutes of interview)**

“Hello. Could I please speak with (candidate’s name). My name is Delara Javat and I am a medical student from James Cook University, conducting a research project with the Mackay Base Hospital (MBH) cardiology department. The project is concerned with patients who were admitted to MBH with heart attacks or chest pain between July 2012- June 2013 and February 2015- January 2016. As such we have noted that you are an eligible candidate. Participation in the project involves answering a few questions over the phone regarding your health after your treatment.

Please note that participation in this study is voluntary. You can consult with others before consenting. If you decide not to participate it will not affect your treatment. If you enrol in the project you maintain the right to withdraw at any time without stating a reason. Upon withdrawing you can choose to retract your supplied information. Deciding to withdraw will not affect your routine treatment.

All information will be de-identified in all future publications of the project.

If you feel uncomfortable at any time and would like to stop the interview, please let me know.

If you have any questions, please feel free to ask.

Is it okay if we proceed with the interview?”

**Introduction (if someone else answers the phone)**

“Hello, could I please speak to (candidate’s name)? My name is Delara Javat and I am a medical student from James Cook University, conducting a research project with the Mackay Base Hospital (MBH) cardiology department. The project is concerned with patients who were admitted to MBH with heart attacks or chest pain between July 2012- June 2013 and February 2015- January 2016. As such we have noted that (candidate name) is an eligible candidate. Participation in the project involves answering a few questions over the phone regarding (candidate name)’s health in after their treatment”.

*The phone would then be handed to the candidate in question and the initial introduction (first 2 mins of the interview) would be re-iterated.*

**Verbal consent**

1. Did patient consent to performing interview via phone?

| - Yes | - No |
| --- | --- |

1. Does patient want to withdraw any already supplied information?

| - Yes | - No |
| --- | --- |

1. Comments

**Project outline**

“Our project is looking at the health of patients after their heart procedures in Mackay Base Hospital”.

**Key questions**

**Q1: “**Have you had to return to hospital after your **angiogram** in Mackay Base Hospital?”

**Q1a**: “If so, could you please tell me what happened?”

**Q1b:** “How many months after your **angiogram** did this occur?”

**Q2:** “Have you had to return to hospital after your stenting procedure in Mackay Base Hospital?

**Q2a**: “If so, could you please tell me what happened?”

**Q2b:** “How many months after your stenting did this occur?”

**Prompts:**

**P1:** “Possible complications from angiograms include, bruising in your arm or groin, a stroke, heart attack, difficulty breathing, emergency heart surgery, treatment for aneurysms”

**P2:** “Possible complications from stenting include, bruising in your arm or groin, abnormal heart beat, heart attack, difficulty breathing, stroke, emergency heart surgery treatment for aneurysms”.

**If speaking to the relative of a deceased patient**

“I am very sorry to hear of your loss. Please accept my apology for raising the matter. I understand it must be distressing for you. Would you like to stop the interview? Is there anything you would like to ask? If you would like, we can refer you to a counselling service run by the Mackay Base Hospital”.

*The relative would then be given the phone number of the allied health social workers, from Mackay Base Hospital, who are able to provide counselling. The relative would also be warmly advised to contact their general practitioner for additional support. The interview will be stopped as per the relative’s wishes.*

**End of the interview**

We will thank the participant for their time.

The participant will be supplied with our contact details if they have any further questions.
