# Supplementary material for: Regional to tertiary inter-hospital transfer versus in-house percutaneous coronary intervention in acute coronary syndrome
Source: PLoS One. 2018 Jun 21;13(6):e0198272. doi: 10.1371/journal.pone.0198272 (PMC6013182; doi:10.1371/journal.pone.0198272)
Supplement: S3 Appendix — A: Cardiology satisfaction survey- patient information leaflet. B: Cardiology satisfaction survey. (DOCX) [file pone.0198272.s003.docx]

**S3A Appendix. Cardiology satisfaction survey patient information leaflet**


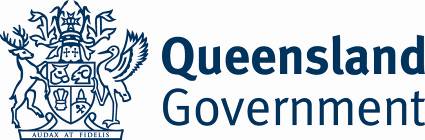
**
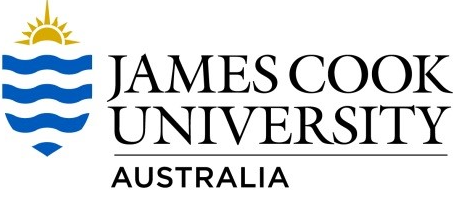
**

**Cardiology Satisfaction Survey: Patient Information Leaflet**

You are invited to take part in a satisfaction survey run by the Cardiology Department of Mackay Base Hospital and James Cook University.

This patient information leaflet explains why the survey is being done and what would be involved if you choose to take part.

**Aim of this satisfaction survey**

This survey seeks to compare the satisfaction levels between patients receiving their angiograms in Mackay Base Hospital with those who were transferred to another hospital for treatment.

**What is an angiogram?**

- This is an investigation which uses dye and X-rays to look for any narrowing of the blood vessels surrounding your heart.

**Why is this survey being conducted?**

- The survey is part of a larger study comparing the differences in **1) health outcomes**, **2)** **satisfaction** and **3)** **cost** between patients receiving treatment in the Cardiology Department in Mackay Base Hospital with those transferred to other hospitals.
- We are comparing information from patients who were treated from **1^st^ July 2012- 30^th^ June 2013** with those who are being treated from **1^st^ February 2015- 31^st^ January 2016.**

**Why have I been chosen?**

- The Cardiology Department in Mackay Base Hospital has noted that you have received an angiogram between 1^st^ July 2012- 30^th^ June 2013 or 1^st^ February 2015- 31^st^ January 2016.
- You are eligible because you were either treated in Mackay Base Hospital or transferred to another hospital for medical care.

**How do I take part?**

If you take part you will need to:

1. Complete the enclosed satisfaction survey. This should take you about **5 minutes**.
2. We ask that you please send the survey back to us in the **reply-paid envelope.**

**Is participation voluntary?**

Yes. Participation is voluntary. If you do not participate your medical care will not be affected.

**Are there any risks in taking part?**

No physical or emotional risks are involved with taking part in this survey.

**What are the possible benefits of this survey?**

Completing this survey will help us understand how satisfied you were with your treatment. This will help us improve the way we look after patients in the Cardiology Department in Mackay Base Hospital.

**Will my information in this study be kept confidential?**

Yes. Your information will only be available to the researchers involved in the study. All written copies will be stored in locked facilities. Publications will include de-identified information. The information you provide will be stored for 10 years after the last publication, in accordance with the Code for Responsible Conduct of Research section 2.1. After this time period, all information will be disposed of safely.

**What will happen to the results of the research study?**

The results will be reviewed by the researchers and the Cardiology Department of Mackay Base Hospital. It is expected that the results will be published in medical journals. A written summary of the research findings and future publications will be available for all those who wish to have a copy.

**Who is organizing and funding the research?**

The research is being organised by the **Cardiology Department in Mackay Base Hospital** and **James Cook University**. This survey is led by cardiologists, Dr. Stefan Buchholz and Dr. Michael Zhang, and a final year medical student from James Cook University. Funding for the study has been approved by the School of Medicine and Dentistry at James Cook University

**What should I do next?**

We hope that you will take part in the study by completing and returning the enclosed satisfaction survey. With your support we believe that we can make a significant contribution towards the care of patients in the Cardiology Department of Mackay Base Hospital.

**Ethical approval**

This project has been reviewed and approved by the **Townsville Hospital and Health Service Human Research Ethics Committee (HREC). If you have any concerns regarding the conduct of this project please contact:**

**Role: HREC chair person**

**Phone: (07) 4433 1440**

**Email: TSV-Ethics-Committee@health.qld.gov.au**

**Contact for further information**

If any information is unclear, or if you would like more information, please contact:

**Name:**  Delara Javat

**Role:** Associate investigator, Honours Research Student James Cook University

**Address:** JCU College of Medicine and Dentistry. PO Box 6314. Mackay QLD 4740.

**Email:** [delara.javat@my.jcu.edu.au](mailto:delara.javat@my.jcu.edu.au)

**Mobile:** 0412 786 320

**S3A Appendix. Cardiology satisfaction survey**


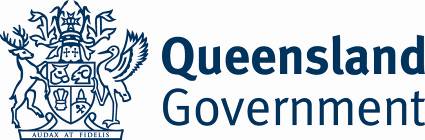

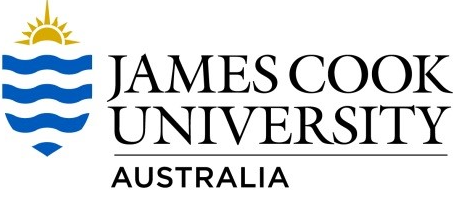


**Cardiology Satisfaction Survey**

The cardiology department at Mackay Base Hospital is **comparing satisfaction** between patients receiving their heart procedures in Mackay Base Hospital with those who were transferred to another hospital for treatment.

Our records suggest that you have had an angiogram. Please take a few minutes to complete this survey and return it to us in the reply paid envelope provided. Your responses are important to us.

Please tick the appropriate box for each question. These questions pertain to the patient to whom this survey was addressed:

1. **Age in years:**

| - 0-9 - 10-19 - 20-29 - 30-39 - 40-49 | - 50-59 - 60-69 - 70-79 - 80-89 - 90-99 |
| --- | --- |

1. **Gender:**

| - Male | - Female |
| --- | --- |

1. **Year of your angiogram**

| - 2012 | - 2013 | - 2014 | - 2015 | - 2016 |
| --- | --- | --- | --- | --- |

1. **Location of your angiogram**

| - Mackay | - Transferred to another hospital |  |
| --- | --- | --- |

1. **If you received your angiogram in Mackay Base Hospital, please answer the following:**

| **How satisfied were you with the following?** | **Very dissatisfied** | **Dissatisfied** | **Neither dissatisfied, nor satisfied** | **Satisfied** | **Very satisfied** |
| --- | --- | --- | --- | --- | --- |
| Length of time spent waiting for your angiogram after being admitted to Mackay Base hospital | 1 | 2 | 3 | 4 | 5 |
| Overall convenience of your angiogram | 1 | 2 | 3 | 4 | 5 |

1. **If you were transferred to another hospital to receive your angiogram, please answer the following:**

| **How satisfied were you with the following?** | **Very dissatisfied** | **Dissatisfied** | **Neither dissatisfied, nor satisfied** | **Satisfied** | **Very satisfied** |
| --- | --- | --- | --- | --- | --- |
| Length of time spent waiting to be transferred to another hospital for your angiogram | 1 | 2 | 3 | 4 | 5 |
| Mode of transport when being transferred to another hospital for your angiogram | 1 | 2 | 3 | 4 | 5 |
| Overall convenience of your angiogram | 1 | 2 | 3 | 4 | 5 |

**7. Comments?**
