# Supplementary material for: Regional to tertiary inter-hospital transfer versus in-house percutaneous coronary intervention in acute coronary syndrome
Source: PLoS One. 2018 Jun 21;13(6):e0198272. doi: 10.1371/journal.pone.0198272 (PMC6013182; doi:10.1371/journal.pone.0198272)
Supplement: S4 Appendix — (DOCX) [file pone.0198272.s004.docx]

**S3 Appendix. Single item analysis from patient satisfaction survey**

**Overall Convenience**

The median score for patient satisfaction with the overall convenience of the angiogram and/or PCI was five (very satisfied) for both groups. The box plots demonstrate a difference in the distribution of the results between groups (S1 Fig.). Excluding outliers the box plot for 2012 to 2013 shows that 50% of the scores were five (very satisfied), 25% of the score were four (satisfied) or below and the lowest score was three (neither dissatisfied nor satisfied). Excluding outliers the box plot for 2015 to 2016 shows that all scores were five (very satisfied) for satisfaction with overall convenience of the angiogram and/or PCI.

The Mann-Whitney U test indicated that the satisfaction with the overall convenience of the angiogram and/or PCI was statistically significantly greater for patients admitted in 2015 to 2016 compared with patients admitted in 2012 to 2013, U = 1957, p <0.05 (two tailed). The sum of ranks was higher for the 2015 to 2016 group (8702) compared with the 2012 to 2013 group (3388).

**Waiting Time**

The median score for patient satisfaction with the length of time spent waiting for the angiogram and/or PCI was five (very satisfied) for both groups. The box plots demonstrate a difference in the distribution of the results between groups (S2 Fig.). The box plot for 2012 to 2013 shows that 25% of the scores were three (neither dissatisfied nor satisfied) or less and the lowest score was one (very dissatisfied). Excluding outliers the box plot for 2015 to 2016 shows that 25% of the scores were four (satisfied) or less and the lowest score was three (neither satisfied nor dissatisfied).

The Mann-Whitney U test indicated that the satisfaction with the time spent waiting for the angiogram and/or PCI was statistically significantly greater for patients admitted in 2015 to 2016 compared with patients admitted in 2012 to 2013, U = 2014, p <0.05(two-tailed). The sum of ranks was higher for the 2015 to 2016 group (8645) compared with the 2012 to 2013 group (3445). The statistical significance was secondary to the different distribution of the scores.

The Mann- Whitney U test results were identical when patient satisfaction scores for overall convenience and the length of time spent waiting for the procedure were compared between patients transferred for treatment and those treated in Mackay.

**Mode of Transport**

The median score for patient satisfaction with the mode of transport was five (very satisfied) for patients transferred to a tertiary hospital between 2012 and 2013. Excluding outliers, the box plot demonstrates that all results for satisfaction with mode of transport were five, very satisfied, (S3 Fig.).

There was no statistically significant relationship between age and gender and patient satisfaction.
