# Supplementary material for: Regional to tertiary inter-hospital transfer versus in-house percutaneous coronary intervention in acute coronary syndrome
Source: PLoS One. 2018 Jun 21;13(6):e0198272. doi: 10.1371/journal.pone.0198272 (PMC6013182; doi:10.1371/journal.pone.0198272)
Supplement: S5 Appendix — (DOCX) [file pone.0198272.s005.docx]

**S5 Appendix. Thematic analysis from patient satisfaction survey**

Comments from the patient satisfaction surveys were provided by 92 patients. We identified five themes: 1) waiting time, 2) inter hospital transfer, 3) interaction with staff, 4) service delivery and 5) patient education. Selected quotations to illustrate each theme are provided in Tablet S5.

**Waiting time**

Patients who were transferred for treatment between 2012 and 2013 were overwhelmed by the length of time spent waiting for angiography. Additionally the separation from family cause anguish for these patients. A subset of patients who were treated in MBH between 2015 and 2016 were conscious of waiting time for angiography. Indeed, patients who did not experience treatment delays at MBH between 2015 and 2016 were impressed with the expedited treatment.

**Inter-hospital transfer**

Patients who were transferred to tertiary hospitals between 2012 and 2013 felt unprepared and were distressed by the logistics surrounding their return travel to Mackay. These patients were also disappointed by the length of time spent waiting for transfer. Patients in both groups expressed gratitude and relief in response to learning that angiography had been made available locally.

**Interaction with hospital staff**

Patients in both groups greatly appreciated the professionalism and caring demeanour of the involved doctors and nurses. Patients unanimously felt reassured by the specialist care they received.

**Service delivery**

Patients who were transferred to tertiary centres largely had a positive experience at the accepting hospital. A subset of patients however did not expect to be given a lower priority and face further treatment delays after they were admitted to the tertiary centre. Patients whose medical information was improperly communicated during the inter hospital transfer were disturbed by the incident because they were concerned for their safety. Those who were treated in MBH from 2015 onwards were impressed by the personable service in hospital and the dedicated follow up.

**Patient education**

Patients treated in MBH felt empowered by the education they received. Those who were kept informed throughout their journey felt reassured during the admission.
