# Supplementary material for: Regional to tertiary inter-hospital transfer versus in-house percutaneous coronary intervention in acute coronary syndrome
Source: PLoS One. 2018 Jun 21;13(6):e0198272. doi: 10.1371/journal.pone.0198272 (PMC6013182; doi:10.1371/journal.pone.0198272)
Supplement: S6 Appendix — (DOCX) [file pone.0198272.s006.docx]

**S6 Appendix. Estimated cost savings from inter-hospital transfer**

Each RFDS fixed wing flight to Townsville was quoted at $3060 per patient, leading to an estimated cost saving per annum of $633 420. The cost of two QAS journeys with a paramedic escort was quoted at $1640. The two journeys included one trip to the Mackay Airport and another to the tertiary accepting hospital in Townsville. Annually, this amounted to a cost saving of $34 468. The cost of escort travel and patient return by car from Townsville was quoted at $233 per patient. This led to an estimated saving per annum of $48 351. Escort accommodation was quoted at $60 per night after the first four nights which were to be paid by the patient. The median length of stay in the accepting hospital was seven days. Therefore accommodation for three days was estimated at $180 per patient. This amounted to an estimated cost saving of $37 260 per annum.
