# Supplementary material for: Regional to tertiary inter-hospital transfer versus in-house percutaneous coronary intervention in acute coronary syndrome
Source: PLoS One. 2018 Jun 21;13(6):e0198272. doi: 10.1371/journal.pone.0198272 (PMC6013182; doi:10.1371/journal.pone.0198272)
Supplement: S1 Fig — (DOCX) [file pone.0198272.s007.docx]

**Figure S1.** **Box plots comparing median patient satisfaction scores for overall convenience between 2012-2013 and 2015-2016**

**
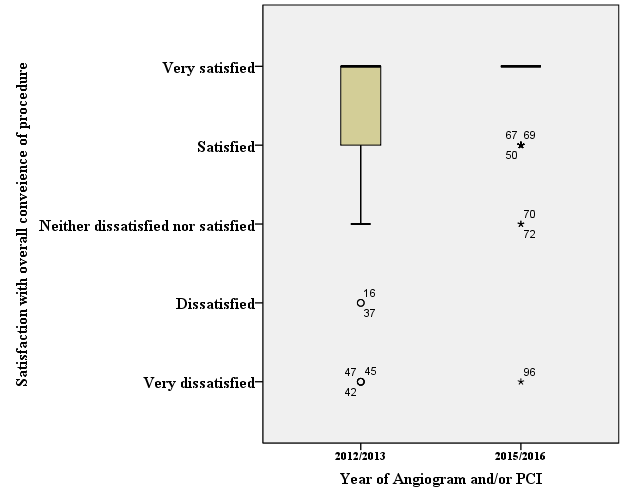
**
