# Supplementary material for: Regional to tertiary inter-hospital transfer versus in-house percutaneous coronary intervention in acute coronary syndrome
Source: PLoS One. 2018 Jun 21;13(6):e0198272. doi: 10.1371/journal.pone.0198272 (PMC6013182; doi:10.1371/journal.pone.0198272)
Supplement: S3 Fig — (DOCX) [file pone.0198272.s009.docx]

**Figure S3:** **Box plot showing the median patient satisfaction score for mode of transport between 2012 and 2013**

**
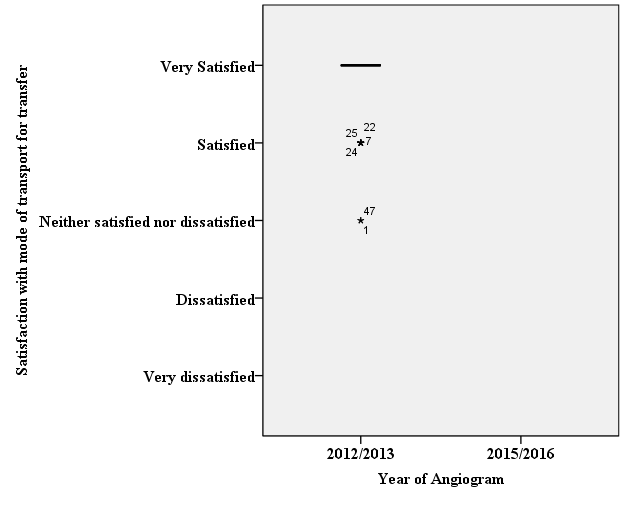
**
