# Supplementary material for: Regional to tertiary inter-hospital transfer versus in-house percutaneous coronary intervention in acute coronary syndrome
Source: PLoS One. 2018 Jun 21;13(6):e0198272. doi: 10.1371/journal.pone.0198272 (PMC6013182; doi:10.1371/journal.pone.0198272)
Supplement: S1 Table — A: TIMI scoring for unstable angina/NSTEMI. B: TIMI scoring for STEMI. C: GRACE scoring for ACS. (DOCX) [file pone.0198272.s010.docx]

**S1A Table. TIMI scoring for unstable angina/NSTEMI**

| **Component** | **Points** |
| --- | --- |
| Age ≥ 65 years | + 1 |
| ≥ 3 coronary artery disease risk factors | + 1 |
| Known coronary artery disease (stenosis ≥ 50%) | + 1 |
| Acetylsalicyclic acid use in past 7 days | + 1 |
| Severe angina (≥2 episodes in 24 hours) | + 1 |
| Electrocardiography ST changes ≥ 0.5mm | + 1 |
| Positive cardiac marker | + 1 |

**S1B Table. TIMI scoring for STEMI**

| **Component** | **Points** |
| --- | --- |
| Age < 65 years | 0 |
| Age 65- 74 years | + 2 |
| Age ≥ 75 years | + 3 |
| Diabetes mellitus or Hypertension or Angina | + 1 |
| Systolic blood pressure <100mmHg | + 3 |
| Heart rate >100 | + 2 |
| Killip Class II- IV | + 2 |
| Weight <67kg (144.4lbs) | + 1 |
| Anterior ST elevation or left bundle branch block | + 1 |
| Time to treatment >4 hours | + 1 |

**S1C Table. GRACE Scoring for ACS**

| **Component** | **Points** |
| --- | --- |
| Age (years) |  |
| Heart rate (bpm) |  |
| Systolic blood pressure |  |
| Creatinine |  |
| Cardiac Arrest at admission | + 1 |
| ST segment deviation on electrocardiogram | + 1 |
| Elevated/abnormal cardiac enzymes | + 1 |
| No congestive heart failure | + 1 |
| Rales and/or jugular vein distension | + 2 |
| Pulmonary oedema | + 3 |
| Cardiogenic shock | + 4 |
