# Supplementary material for: Regional to tertiary inter-hospital transfer versus in-house percutaneous coronary intervention in acute coronary syndrome
Source: PLoS One. 2018 Jun 21;13(6):e0198272. doi: 10.1371/journal.pone.0198272 (PMC6013182; doi:10.1371/journal.pone.0198272)
Supplement: S2 Table — PCI–Percutaneous coronary intervention. CABG–Coronary artery bypass graft surgery. GRACE–Global registry of acute coronary events. TIMI- Thrombolysis in myocardial infarction. (DOCX) [file pone.0198272.s011.docx]

**Table S2. Multivariate logistic regression for the primary outcome - composite of all-cause mortality, recurrent MI and recurrent ischaemia at 6 months**

| **Independent variables** | **Number Analysed** | **Univariate Logistic Regression** | | **Multivariate Logistic Regression** | |
| --- | --- | --- | --- | --- | --- |
|  |  | OR (95% CI) | P value | OR (95% CI) | P value |
| Age | 424 | 0.99 (0.97- 1.03) | 0.84 | 1.0 (0.95- 1.1) | 0.94 |
| Male | 424 | 1.1 (0.49- 2.3) | 0.89 | 0.87 (0.37-2.1) | 0.75 |
| Hypertension | 422 | 1.3 (0.61 – 2.9) | 0.47 | 0.76 (0.29 – 1.9) | 0.57 |
| Diabetes | 423 | 0.86 (0.37- 1.9) | 0.73 | 1.4 (0.52- 3.9) | 0.48 |
| Smoker | 422 | 0.81 (0.39- 1.7) | 0.57 | 0.95 (0.38 – 2.4) | 0.92 |
| Previous myocardial infarction | 423 | 0.81 (0.3- 2.2) | 0.67 | 1.7 (0.45 – 6.7) | 0.42 |
| Previous PCI | 424 | 0.81 (0.27- 2.4) | 0.69 | 0.78 (0.21 – 2.9) | 0.70 |
| Previous CABG | 423 | 2.1 (0.75- 5.8) | 0.16 | 0.38 (0.10- 1.5) | 0.16 |
| Time to procedure | 391 | 1.0 (0.91- 1.1) | 0.83 | 1.1 (0.94- 1.2) | 0.32 |
| Year of admission | 424 | 1.1 (0.55- 2.3) | 0.75 | 0.71 (0.24 – 2.1) | 0.54 |
| GRACE score | 386 | 0.99 (0.98- 1.0) | 0.26 | 0.99 (0.97 – 1.0) | 0.18 |
| Past stroke | 422 | 1.2 (0.15- 9.9) | 0.85 | 0.69 (0.08- 6.2) | 0.74 |
| ≥ 3 vessels with ≥ 70% stenosis | 424 | 0.49 (0.15- 1.7) | 0.26 | 1.2 (0.31 – 4.9) | 0.76 |
| Clopidogrel loading | 393 | 0.65 (0.28-1.5) | 0.32 | 0.44 (0.15- 4.9) | 0.13 |
| Ticagrelor loading | 399 | 1.1 (0.46-2.5) | 0.88 | 0.65 (0.23- 1.9) | 0.42 |
| Hyperlipidaemia | 422 | 1.7 (0.76- 3.9) | 0.17 |  |  |
| Chronic Kidney Disease | 422 | 0.97 (0.22- 4.3) | 0.97 |  |  |
| Body mass index | 377 | 1.1 (0.99- 1.1) | 0.07 |  |  |
| Positive family history | 417 | 0.87 (0.41- 1.9) | 0.71 |  |  |
| TIMI score | 409 | 1.0 (0.76- 1.3) | 0.94 |  |  |

**PCI –** Percutaneous coronary intervention

**CABG –** Coronary artery bypass graft surgery

**GRACE –** Global registry of acute coronary events

**TIMI**- Thrombolysis in myocardial infarction
