# Supplementary material for: Regional to tertiary inter-hospital transfer versus in-house percutaneous coronary intervention in acute coronary syndrome
Source: PLoS One. 2018 Jun 21;13(6):e0198272. doi: 10.1371/journal.pone.0198272 (PMC6013182; doi:10.1371/journal.pone.0198272)
Supplement: S3 Table — PCI–Percutaneous coronary intervention. CABG–Coronary artery bypass graft surgery. GRACE–Global registry of acute coronary events. TIMI- Thrombolysis in myocardial infarction. (DOCX) [file pone.0198272.s012.docx]

**Table S3.** **Multivariate logistic regression for the secondary outcome - composite of all-cause mortality, recurrent MI and recurrent ischaemia at 1 month**

| **Independent variables** | **Number Analysed** | **Bivariate analysis** | | **Binomial Logistic Regression** | |
| --- | --- | --- | --- | --- | --- |
|  |  | OR (95% CI) | P value | OR (95% CI) | P value |
| Age | 425 | 1.01 (0.95- 1.1) | 0.76 | 1.1 (0.93 – 1.2) | 0.37 |
| Male | 425 | 1.5 (0.33- 6.8) | 0.59 | 0.95 (0.14 – 6.4) | 0.96 |
| Hypertension | 423 | 1.5 (0.29- 7.8) | 0.63 | 0.86 (0.12- 6.3) | 0.88 |
| Diabetes | 424 | 1.9 (0.44- 9.0) | 0.37 | 0.52 (0.07 – 4.0) | 0.53 |
| Smoker | 423 | 1.6 (0.36- 7.4) | 0.54 | 0.32 (0.04- 2.9) | 0.31 |
| Previous myocardial infarction | 424 | 0.72 (0.09- 6.1) | 0.77 | 8.5 x 10 – 0.00) | 1.0 |
| Previous PCI | 425 | 2.4 (0.45- 12.0) | 0.31 | 0.14 (0.01 – 1.3) | 0.09 |
| Previous CABG | 424 | 1.8 (0.21- 15) | 0.60 | 0.32 (0.02 – 6.2) | 0.45 |
| Time to procedure | 392 | 1.0 (0.85- 1.2) | 0.78 | 1.0 (0.76- 1.4) | 0.92 |
| Year of admission | 425 | 0.66 (0.15- 2.9) | 0.58 | 1.2 (0.11 – 13.1) | 0.87 |
| GRACE score | 387 | 0.99 (0.97- 1.0) | 0.76 | 0.98 (0.93 – 1.0) | 0.43 |
| Past stroke | 423 | 3.6 x 10^-8^ (0.00) | 0.99 | 8.3 x 10^6^ (0.00) | 1.0 |
| ≥ 3 vessels with ≥ 70% stenosis | 425 | 0.82 (0.09- 6.9) | 0.85 | 1.9 (0.12 – 30.3) | 0.66 |
| Clopidogrel loading | 395 | 0.89 (0.16-4.9) | 0.89 | 0.2 (0.02 – 2.6) | 0.22 |
| Ticagrelor loading | 401 | 0.54 (0.12 – 2.4) | 0.42 | 0.17 (0.02 – 1.5) | 0.12 |
| Hyperlipidaemia | 423 | 0.89 (0.2- 4.0) | 0.87 |  |  |
| Chronic Kidney Disease | 423 | 3.4 x 10^-8^ (0.00) | 0.99 |  |  |
| BMI | 378 | 1.05 (0.94- 1.2) | 0.40 |  |  |
| Positive family history | 418 | 0.28 (0.03- 2.3) | 0.23 |  |  |
| TIMI score | 410 | 1.2 (0.69- 2.1) | 0.51 |  |  |

**PCI –** Percutaneous coronary intervention

**CABG –** Coronary artery bypass graft surgery

**GRACE –** Global registry of acute coronary events

**TIMI**- Thrombolysis in myocardial infarction
