# Supplementary material for: Regional to tertiary inter-hospital transfer versus in-house percutaneous coronary intervention in acute coronary syndrome
Source: PLoS One. 2018 Jun 21;13(6):e0198272. doi: 10.1371/journal.pone.0198272 (PMC6013182; doi:10.1371/journal.pone.0198272)
Supplement: S4 Table — PCI–Percutaneous coronary intervention. (DOCX) [file pone.0198272.s013.docx]

**Table S4. Patient characteristics from satisfaction surveys**

| **Variable** | **All patients (n =156)** | **2012-2013 (n = 53)** | **2015-2016 (n = 103)** |
| --- | --- | --- | --- |
| Location angiogram and/or PCI, n (%) |  |  |  |
| Transferred | 53 (34) | 53 (100) | 0 |
| Mackay | 103 (66) | 0 | 103 (100) |
| Age categories, n (%) |  |  |  |
| 40- 49 | 7 (4.5) | 1 (1.9) | 6 (5.8) |
| 50- 59 | 31 (20) | 8 (15) | 23 (22) |
| 60- 69 | 56 (36) | 24 (45) | 32 (31) |
| 70- 79 | 40 (26) | 13 (25) | 27 (26) |
| 80- 89 | 22 (14) | 7 (13) | 15 (15) |
| Males, n (%) | 102 (65) | 36 (68) | 66 (64) |

**PCI** – Percutaneous coronary intervention
