# Supplementary material for: Regional to tertiary inter-hospital transfer versus in-house percutaneous coronary intervention in acute coronary syndrome
Source: PLoS One. 2018 Jun 21;13(6):e0198272. doi: 10.1371/journal.pone.0198272 (PMC6013182; doi:10.1371/journal.pone.0198272)
Supplement: S5 Table — IR–Interquartile range. N/A–Not applicable. (DOCX) [file pone.0198272.s014.docx]

**Table S5. Summated score analysis and individual score analysis of patient satisfaction surveys**

| **Variable** | **All patients (n = 156)** | **2012-2013 (n = 53)** | **2015-2016 (n = 103)** |
| --- | --- | --- | --- |
| Summated patient satisfaction score, median (IR) | 10 (2.0) | 9.0 (3) | 10 (1) |
| Satisfaction with length of time spent waiting for angiogram and/or PCI, median (IR) | 5.00 (1) | 5.0 (2) | 5.0 (1) |
| Satisfaction with overall convenience, median (IR) | 5.0 (1) | 5.0 (1) | 5.0 (0) |
| Satisfaction with mode of transport, median (IR) | 5.0 (3) | 5.0 (3) | N/A |

**IR** – Interquartile range

**N/A** – Not applicable
