# Supplementary material for: Regional to tertiary inter-hospital transfer versus in-house percutaneous coronary intervention in acute coronary syndrome
Source: PLoS One. 2018 Jun 21;13(6):e0198272. doi: 10.1371/journal.pone.0198272 (PMC6013182; doi:10.1371/journal.pone.0198272)
Supplement: S6 Table — (DOCX) [file pone.0198272.s015.docx]

**Table S6. Illustrative quotations supporting thematic analysis of patient satisfaction surveys**

| **Themes** | **Number analysed, n (%)** | **Quotations for patients transferred 2012- 2013** | **Quotations for patients treated in MBH 2015- 2016** |
| --- | --- | --- | --- |
| **Waiting time for procedure** | 7 (4.5) | “2 days waiting in Emergency for a bed.”  “Had to wait over a week to have angio... “  “6 hour wait for angiogram once prepped.”  “I had to lay around in a hospital all weekend 400 kms away from my family.... On the following Friday, one week later, I finally had it done...absolutely pathetic.” | “Admitted Monday but could not have procedure until late Thursday.”  “Was admitted on Friday night and did not have my angiogram until the following Monday afternoon.”  “I am very satisfied with treatment and the short wait time. Also I am very impressed with everything at MBH.”  “No waiting period whatsoever for the angiogram.” |
| **Inter hospital transfer** | 11 (7.1) | “Angiography not available in Mackay at the time. Happy that it can now be done here and would prefer to stay in Mackay if I could.”  “Had to wait days before being transferred and had to arrange to be picked up after treatment.”  “I was transferred to the Townsville hospital 4-5 days later.”  “...had to leave my disabled wife.”  “Time to wait for transport could have been quicker.”  “Had to borrow clothes to get home.”  “Had to be transferred for the procedure and then released to find my own way back to Mackay; was very disappointing.”  “Pleased we now can have this done in Mackay as it always an inconvenience for family when you have to travel for care.” | “Very happy to be able to receive angiography locally and not have to travel to Townsville”.  “Made me feel reassured that I did not have to travel for treatment.” |
| **Interaction with hospital staff** | 32 (21) | “Overall I was treated very well by all involved.”  “.. After care by specialist at Mackay was second to none.  “I found the doctors and nurses at the Townsville hospital absolutely wonderful.” | “Would like to extend my thanks to all staff of MBH for treatment and care. They are truly professional and caring.”  “Staff were very professional and compassionate.”  “Cannot think of a single incident from entry to departure, from the cleaner to the specialist and everyone in between....Never been so well looked after in my life.”  “Highest praise and regard for all staff. Professionalism at MBH was excellent. Best public hospital in Queensland.”  “Always treated with respect and kindness.”  “All doctors and nurses [were] excellent. Could not have been in better hands.” |
| **Service delivery** | 39 (25) | “Great system of care for when you are high risk patient. Very happy with the outcome and can now continue with my bucket list. RFDS and Base are great.”  “Experience at Mackay and Townsville was very good...”  “In all the whole service was great.”  “The care I received in Mackay and Townsville was excellent.”  “Well looked after.”  “All good service in Townsville and Mackay.”  “My husband and I were completely traumatised with what was happening to him and certainly didn’t need any of this farcical carry on with mistaken/mixed up files.”  “...and then be told each day from Monday that there was someone more important than me to have an angiogram.” | “Treatment and attention in the cardiac ward in Mackay was first class.”  “Great personable service, well explained, felt very safe and well looked after.”  “Very organised and well done.”  “Received nothing but the best care from all concerned. Very fortunate to have wondering cardiac care in Mackay now. Wonderful care and follow up.”  “Cannot fault the care I received at MBH cardiac unit...”  “Have never been in a hospital before and felt like I was treated like royalty.”  “Could not have asked for better care.”  “Was very impressed with the overall medical services at the MBH.” |
| **Patient education** | 3 (1.9) | None available | “CCU staff ...helped with questions and concerns.”  “All a very smooth process, couldn’t fault it. Everything was explained and questions answered promptly so I understood everything...”  “Answered all my questions in a reassuring manner.” |
